# Systems metabolic engineering of glutathione biosynthesis in *Saccharomyces cerevisiae*: Pathway balancing coupled with enzyme screening for high-titer production

**DOI:** 10.1016/j.engmic.2025.100243

**Published:** 2025-09-23

**Authors:** Zhiqi Hu, Mengyuan Su, Qibing Liu, Ying Li, Yunxiang Liang, Shuangquan Li, Yingjun Li

**Affiliations:** a National key laboratory of agricultural microbiology and College of Life Science and Technology, Huazhong Agricultural University, Wuhan 430070, China; b Hubei Lucnova Bio-Technology Co., Ltd, Huanggang 438000, China

**Keywords:** Glutathione biosynthesis, *Saccharomyces cerevisiae*, Enzyme fusion, CRISPR/Cas9, Bioreactor scale-up

## Abstract

Glutathione (GSH), an essential tripeptide thiol critical for cellular redox regulation, has significant value in the pharmaceutical and nutraceutical industries. To overcome limitations of traditional GSH extraction methods, this study established a microbial cell factory platform in *Saccharomyces cerevisiae* through integrated metabolic engineering strategies: (1) host strain screening identified NJ-SQYY with superior GSH accumulation (74.14 mg·L⁻¹, 8.27 mg·g^-1^ dry cell weight [DCW]); (2) CRISPR/Cas9-mediated genomic integration of bacterial *gshAB* introduced with a bifunctional glutathione synthetase; (3) systematic optimization via promoter tuning and Gsh1-Gsh2 enzyme fusion, and *CYS3* overexpression-resolved γ-glutamylcysteine bottlenecks. These interventions synergistically enhanced GSH synthesis to 339.3 mg·L⁻¹ in shake flasks (4.6-fold increase), representing the highest reported titer in chromosomally engineered *S. cerevisiae*. Scaling to dissolved oxygen-coupled fed-batch fermentation in a 5-L bioreactor produced 997.46 mg·L⁻¹ GSH at 33.85 mg·g⁻¹ DCW. This study demonstrated a holistic metabolic engineering-to-bioprocessing approach for industrial GSH biomanufacturing.

## Highlights

• Novel plasmid-free S. cerevisiae produces 997.46 mg·L⁻¹ glutathione.
• Gsh1-Gsh2 fusion resolves γ-glutamylcysteine bottleneck in GSH pathway.
• DO-coupled fed-batch boosts GSH titer 2.9 × vs. flasks in 5-L bioreactor.

## Graphical abstract

**Figure:**
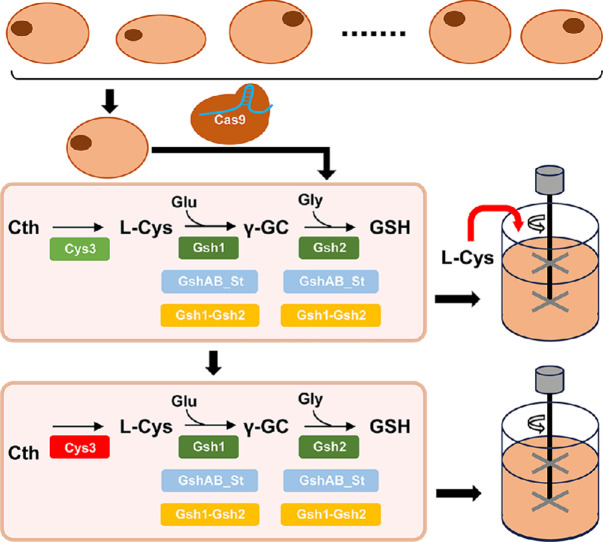

## 1 Introduction

Glutathione (γ-glutamyl-cysteinyl-glycine, GSH), a ubiquitous tripeptide thiol, exists in two redox states, reduced monomeric (GSH) and oxidized dimeric (GSSG), which is connected through a disulfide bridge [20]. This evolutionarily conserved metabolite plays pivotal roles in cellular redox homeostasis, xenobiotic detoxification, immune modulation, and the regulation of proliferation and apoptosis pathways in eukaryotes and prokaryotes [1,24]. The expanding application of GSH in pharmaceutical formulations, nutraceutical additives, and cosmetic products has driven the demand for cost-effective microbial production systems [3,25].

Microbial biosynthesis has distinct advantages over plant extraction and chemical synthesis, owing to its superior scalability and process economics [2]. Although both *Escherichia coli* and yeast have been engineered for GSH production, yeast systems demonstrate superior industrial compatibility through enhanced product tolerance and GRAS (Generally Recognized as Safe) [16,17]. Moreover, GSH is an intracellular product of yeast, and its production can be enhanced in two ways: by increasing cell biomass (high-cell-density culture techniques) or increasing GSH content in yeast; increasing cell biomass is easier using fermentation techniques than by increasing intracellular GSH content [26,34]. In addition, increased intracellular GSH levels facilitate downstream processes. The canonical GSH biosynthetic pathway in eukaryotes involves two ATP-dependent enzymatic steps: γ-glutamylcysteine formation by glutamate-cysteine ligase (GCL, encoded by *GSH1*) followed by glycine addition via glutathione synthetase (GS, *GSH2*) [37]. Overexpression of the endogenous *GSH1* and *GSH2* genes in *Saccharomyces cerevisiae* resulted in a 1.5-fold increase in glutathione (GSH) content compared to that in the wild-type strain [7]. In addition, by introducing Gsh1 and Gsh2, which are derived from *S. cerevisiae*, into *Pichia pastoris*, the production of GSH in shake flask cultures reached 217 mg·L⁻¹ [5]. Notably, certain Gram-positive bacteria employ a bifunctional glutathione synthetase enzyme (GshAB/F) that catalyzes both reactions through distinct N-terminal GCL-like and C-terminal ATP-grasp domains, circumventing the feedback inhibition imposed on eukaryotic GCL by GSH [18,31]. Therefore, heterologous expression of GshAB/F provides the possibility for efficient GSH synthesis. The majority of heterologous GshAB/F expression studies have been conducted using *E. coli* GshF derived from *Streptococcus thermophilus,* which is heterologously expressed in *E. coli* to synthesize GSH, and remarkable results have been achieved by many researchers [12,15,32]. The heterologous expression of GshF from *Lactobacillus* spp. (e.g., *Lactobacillus plantarum*) in *E. coli* robustly enhanced glutathione biosynthesis, achieving a 177.9 % yield increase under optimized conditions while suppressing the expression of native GSH-related genes, establishing GshF as a potent metabolic engineering tool [38]. In a recent study, GshFAp and GshFAs from *Actinobacillus* spp. exhibited high end-product inhibition resistance and dimeric conformations, enabling recombinant *E. coli* to produce 34.1 mM and 36.6 mM glutathione with >85 % molar yield from L-cysteine, supported by conserved substrate-binding motifs for industrial biosynthesis [39]. To enhance glutathione (GSH) biosynthesis in *S. cerevisiae*, researchers established a dual engineering strategy: (1) implementing the GshFAp-dependent secondary pathway and (2) introducing the Pro1 (γ-glutamyl kinase)- and GshB (GS)-mediated tertiary pathway, achieving a high GSH titer of 216.50 mg L⁻¹ [28]. However, the prokaryotic origin of GshAB/F enzymes raises potential protein-folding challenges in eukaryotic expression systems. This limitation can be addressed by co-expressing the *E. coli* GroEL/ES chaperonin system, a well-characterized prokaryotic molecular chaperone complex that facilitates protein folding [8]**.** Experimental evidence has demonstrated that the co-expression of cognate GroEL/ES chaperonins in *S. cerevisiae* enables the functional rescue of otherwise impaired heterologous xylose isomerase and arabinose isomerase through proper protein folding [29,35]. Researchers have successfully utilized the GroEL/ES chaperonin system to enable the functional expression of *Pseudomonas denitrificans* and *Rhodospirillum rubrum* RuBisCO in *S. cerevisiae*, thereby redirecting metabolically generated CO₂ into glycerol 3-phosphate biosynthesis through carbon fixation and ultimately enhancing ethanol flux [22]. Recent validation studies have demonstrated the successful heterologous expression of GshAB_Ap in *S. cerevisiae* through GroEL/ES co-chaperoning, confirming the technical feasibility of this approach [11].

In addition to the genes directly involved in GSH synthesis, the expression of genes involved in related pathways such as precursor metabolic pathways and antioxidant systems also affected GSH synthesis. The overexpression of sulfur assimilation genes (*MET14* and *MET16*) increased GSH titers by up to 1.2-fold and 1.4-fold, respectively [7]**.** Strategic overexpression of key enzymes in the L‑serine biosynthetic pathway, *SER1, SER2, SER3*, and *SER33*, enhanced GSH production in *S. cerevisiae* by 1.3-, 1.4-, 1.9-, and 1.9-fold, respectively. Notably, the combinatorial overexpression of *SHM2* and *CYS4* achieved maximal GSH titers of 64 mg·L^-1^, representing a 2.5-fold increase over the parental strain [14]. These results demonstrate that pathway engineering through precursor (L‑serine/L-cysteine) flux enhancement is an effective strategy for GSH overproduction.

Multi-omics interrogation of GSH-overproducing *S. cerevisiae* strains uncovered a redox-mediated regulatory axis, wherein the attenuation of respiratory complex III elevated ROS levels, activating *SKN7*- and *YAP1*-dependent stress adaptation pathways that transcriptionally upregulate glutathione biosynthesis [41]. The transcription factor Yap1 orchestrates oxidative stress adaptation in *S. cerevisiae* by driving the expression of antioxidant defense systems, thereby maintaining redox homeostasis within physiological thresholds [6]. Transcriptional activation through *YAP1* overexpression elevated both GSH biosynthesis (*GSH1/2*) and cysteine metabolism (*CYS3/4*) genes, achieving 32.5 mg·L⁻¹ production [21].

This study established a systematic engineering framework for microbial GSH biosynthesis through three sequential phases: (1) chassis selection: phenotypic screening identified high-performance yeast strains with intrinsic redox homeostasis advantages; (2) enzyme optimization: phylogenetic analysis guided the selection of bacterial GshAB isoforms, which were subsequently enhanced via a combinatorial optimization strategy comprising codon adaptation, promoter tuning, and plasmid copy number control; and (3) metabolic rewiring: endogenous pathway augmentation was achieved through Gsh1-Gsh2 fusion constructs, coupled with precursor flux amplification via L-cysteine biosynthesis pathway engineering. Although concerted efforts have been made to synchronize precursor availability and redox balance through Yap1 transcriptional activator overexpression and prokaryotic chaperone (GroEL/ES) co-expression, these interventions have demonstrated suboptimal coordination in the eukaryotic host. Nevertheless, our multidimensional approach not only established a robust GSH production platform (the shaking flask level reached 339.3 mg·L^-1^) with industrial scalability, but also provided mechanistic insights into three critical aspects of microbial metabolic engineering: (i) host-pathway biocompatibility requirements, (ii) cross-kingdom enzyme adaptation barriers, and (iii) metabolic flux-redox homeostasis crosstalk.

## 2 Materials and methods

### 2.1 Genes and their sources

The *gshAB* genes of nine organisms: *Actinobacillus pleuropneumoniae, Lactobacillus casei, Lactobacillus rhamnosus, Lactobacillus plantarum, Lactobacillus paracasei, Enterococcus faecalis, Enterococcus faecium, Streptococcus thermophilus*, and *Clostridium perfringens* (Table S5), were derived. The nine promoters and the *YAP1, GSH1, GSH2, CYS3, CYS4, SER3, MET16*, and *SHM2* genes originated from *S. cerevisiae* NJ-SQYY. The *groS* and *groL* (*groSL*) genes were obtained from *E. coli* DH5α.

### 2.2 Culture medium and culture conditions

The yeast extract peptone dextrose (YPD) medium, composed of 20 g·L⁻¹ glucose, 20 g·L⁻¹ peptone, and 10 g·L⁻¹ yeast extract, was utilized for yeast screening, cultivation, and fermentation seed preparation. The fermentation medium, containing 25 g·L⁻¹ sucrose, 28 g·L⁻¹ yeast extract, 7 g·L⁻¹ tryptone, 2 g·L⁻¹ (NH_4_)_2_SO_4_, 1 g·L⁻¹ KH_2_PO_4_, 1 g·L⁻¹ MgSO_4_, and 2 g·L⁻¹ NaCl, was employed for the fermentation of engineered yeast strains in shake flasks and fermenters. The Luria–Bertani (LB) medium, consisting of 5 g·L⁻¹ yeast extract, 10 g·L⁻¹ tryptone, and 10 g·L⁻¹ NaCl, was used for *E. coli* DH5α culture. All solid media were prepared by supplementing the respective base media with 15 g·L⁻¹ agar.

For yeast cultures, static culture conditions were maintained at 28 °C, whereas shaking culture conditions were set at 28 °C and 200 rpm. For *E. coli* DH5α cultures, the static culture temperature was 37 °C, and the shaking culture conditions were set at 37 °C and 180 rpm. Additionally, 100 μg·mL⁻¹ ampicillin was added to the *E. coli* culture medium to ensure stable plasmid inheritance.

### 2.3 Validation of antibiotic resistance screening markers

Using the plate coating method, a concentration gradient of 50 μg·mL⁻¹ was established within the range of 0 to 250 μg·mL⁻¹ to determine the minimum inhibitory concentration (MIC) of the antibiotic that suppresses the growth of the strain. The bacterial suspension, transformed with a plasmid harboring an antibiotic resistance gene, was spread onto agar plates containing the designated antibiotic concentrations. Following incubation for 48 h, single colonies were isolated and subjected to PCR verification using plasmid-specific primers.

### 2.4 Plasmid construction

The insertion region was selected from the *S. cerevisiae* S288C genome available in the National Center for Biotechnology Information database. The spacer was chosen using the Sequence Scan for CRISPR (http://crispr.dfci.harvard.edu/SSC). Two single-stranded spacers with vector homology sequences were synthesized, mixed in a 1:1 ratio, and annealed. The plasmid p416-sgRNA-NeoR was digested with *Bsa*I, ligated to the spacer using T4 DNA ligase, and named p416-SN-SP. This plasmid was PCR-linearized, and 500-bp homologous arms were amplified from the NJ-SQYY genome. Homologous recombination between the linear plasmid and arms produced p416-SN-SP(HA). The NJ-SQYY promoter and terminator were amplified with the *Bsa*I sites, fused via PCR, and cloned into a T vector to create pCE2-P-T. The target gene, amplified with the *Bsa*I sites, was digested with *Bbs*I and *BsaI and* ligated into pCE2-P-T to produce pCE2-P-gene-T. Linearizing p416-SN-SP(HA) and amplifying P-gene-T allowed for homologous recombination to generate the final plasmid, p416-SN-gene-SP.

### 2.5 Competent cell preparation and plasmid transformation

Preparation of competent cells and subsequent transformation of *S. cerevisiae*: A Super Yeast Competence Preparation and Transformation Kit (SK2401, Coolaber) was used.

Preparation of chemically competent *E. coli* DH5α cells: Competent cells were prepared via calcium chloride treatment with the following modifications: fresh colonies from glycerol stocks were streaked onto LB agar and incubated overnight at 37 °C. A single colony was inoculated into 10 mL LB broth in a 250 mL baffled flask and grown aerobically (200 rpm, 37 °C) for 12–16 h. The culture was subcultured (1 % inoculum) in 100 mL of fresh LB medium until the mid-log phase (OD_600_ = 0.35), then the cells were harvested via centrifugation (3000 × *g*, 10 min, 4 °C) and sequentially treated with ice-cold 0.05 M CaCl₂. This initial resuspension was followed by 30 min of incubation on ice, then secondary centrifugation under identical conditions. The final pellet was suspended in 4 mL of a cryoprotectant solution (0.05 M CaCl₂, 15 % glycerol) and aliquoted (100 μL) for flash-freezing at −80 °C.

*E. coli* transformation procedure: Competent cells were thawed on ice (10 min) and gently mixed with 10 μL of a ligation mixture (ligated or recombinant plasmid). Following 30 min of incubation on ice, heat shock was induced at 42 °C for 90 s with immediate recovery for 2 min on ice. The cells were then resuscitated in 700 μL LB medium (180 rpm, 37 °C, 45 min), pelleted (4000 × *g*, 3 min), and resuspended in 100 μL of the supernatant before plating on LB agar containing 100 μg·mL⁻¹ ampicillin. Transformants were selected after 16 h of incubation at 37 °C.

### 2.6 GSH analysis

The GSH concentrations were determined using the 5,5′-dithiobis-(2-nitrobenzoic acid) (DTNB) colorimetric method. Briefly, calibration standards (0–100 mg·L⁻¹) were prepared by sequentially diluting a 0.2 g·L⁻¹ GSH stock solution with ddH₂O to a final volume of 2 mL. Each standard was pre-incubated with 3 mL of Tris–HCl buffer (pH 8.2), followed by 1 mL of 3 % formaldehyde for 2 min. Reactions were initiated with 5 mL of a DTNB solution (pre-equilibrated to 25 °C) and incubated at 25 °C for 10 min. The absorbance at 412 nm was measured against a ddH₂O blank using a UV–Vis spectrophotometer, with standard curves generated using least-squares regression of concentration-absorbance pairs.

For biological samples, yeast cultures were harvested post-fermentation via centrifugation (5000 × *g*, 5 min, 4 °C). Cell pellets were subjected to two ddH_2_O washes before ethanol extraction (1.5 mL 40 % ethanol, 25–30 °C, 2–3 h with agitation). Clarified supernatants obtained through centrifugation (7000 × *g*, 10 min, 4 °C) were diluted 1:1 with ddH₂O for analysis. Sample absorbance values were converted to GSH concentrations using the calibration equation, enabling the calculation of intracellular GSH yield (mg·g⁻¹ DCW) and cellular content (mg·L⁻¹).

### 2.7 Scale-up in 5-L bioreactors

The composition of the fermentation medium mirrored that of the shake flask culture, containing 40 g·L⁻¹ sucrose, 32 g·L⁻¹ yeast extract, and 8 g·L⁻¹ tryptone. Batch fermentation was conducted in a 2.5 L working volume bioreactor under controlled conditions: the temperature was maintained at 28 °C, the aeration rate was 3 L/min, and dissolved oxygen (DO) levels were sustained at 25 % through agitation speed adjustment. A seed culture (24-h growth phase) was inoculated following standard aseptic protocols. A fed-batch supplementation strategy was initiated at 12 h post-inoculation, with continuous feeding of a concentrated substrate solution (12.5 mL per 30-minute interval) to maintain metabolic flux balance.

### 2.8 Statistical analysis

Experimental data were analyzed using SPSS Statistics software (R26.0.0.0, 64-bit version), with three biological replicates per experimental group. The normality and homogeneity of variance assumptions were verified using appropriate tests. For datasets satisfying variance homogeneity, a one-way ANOVA was performed, followed by Duncan's multiple comparison test as a post-hoc analysis. Statistical significance (*p* < 0.05) was determined through this hierarchical testing approach, with results graphically represented using the letter-coding notation [(A, B, C) or (A, B, C)] to denote significant differences between the groups. Pairwise comparisons were conducted using two-tailed independent Student’s *t*-tests, with significance thresholds defined as follows: *p (^#^*p*) < 0.05, **p (^##^*p*) < 0.01, and ***p (^###^*p*) < 0.001. All numerical data are presented as the mean ± standard deviation.

## 3 Results and discussion

### 3.1 Strain isolation and characterization

**Fig. 1:**
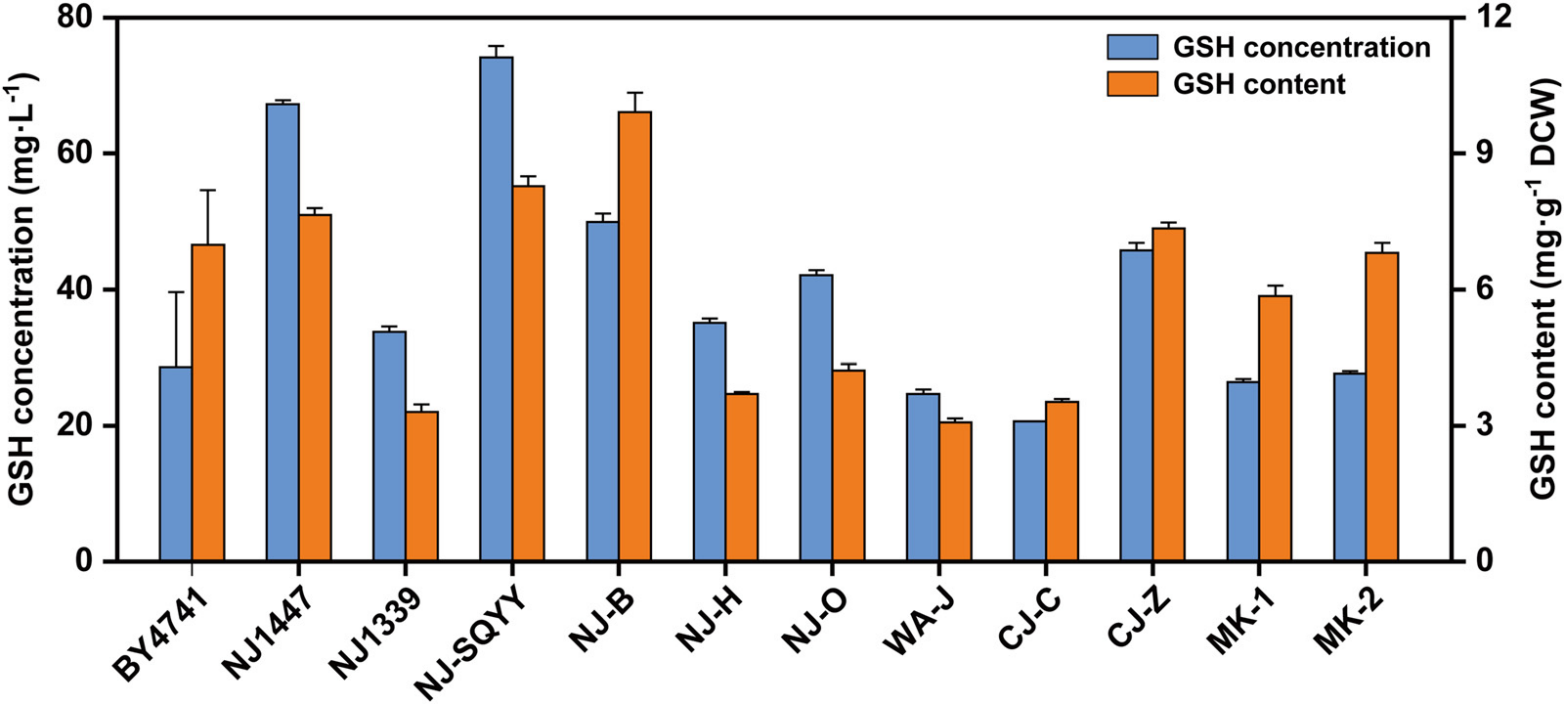
Comparison of fermented glutathione levels in multiple yeast strains. All strains were cultured in YPD medium for 48 h. Data represent the mean ± SD of triplicates.

To expand the yeast biodiversity, we collected microbial samples from distiller grains, vinegar residues, and culture starters from diverse geographical regions. Following enrichment culture and taxon-specific isolation, 31 yeast strains representing 24 distinct species were identified (Table S2). Initial screening revealed elevated GSH levels in *S. cerevisiae, Kluyveromyces marxianus*, and *Candida utilis*, while *Wickerhamomyces anomalus* exhibited superior biomass accumulation. Eleven representative strains from these four species, along with their laboratory-maintained counterparts, were subjected to culture analysis, using BY4741 as the control strain. As illustrated in Fig. 1, strain NJ-SQYY demonstrated the highest GSH titer (74.14 mg·L^-1^, 8.27 mg·g^-1^ DCW), whereas NJ-B showed peak cellular GSH content (9.92 mg·g^-1^ DCW) but lower productivity (49.92 mg·L^-1^). Based on the volumetric yield, NJ-SQYY was selected as the metabolically engineered chassis.

### 3.2 CRISPR/Cas9-mediated genomic integration of Actinobacillus pleuropneumoniae gshAB_Ap enhances glutathione biosynthesis

To establish selection markers for wild-type NJ-SQYY lacking auxotrophic markers, antibiotic susceptibility was systematically evaluated. The MICs of 100 μg·mL^-1^ geneticin (G418) and 150 μg·mL^-1^ hygromycin B (HygB) were determined for complete growth suppression (Fig. S1A–S1B). The shuttle plasmids p415-Cas9-HygR and p416-sgRNA-NeoR, encoding the respective resistance genes, were constructed and transformed into NJ-SQYY cells. The transformants exhibited robust growth on YPD agar containing the two antibiotics (Fig. S1C). The colony PCR verification of eight randomly selected clones confirmed 100 % plasmid retention (Fig. S1D–S1E), validating the efficacy of the HygB/G418 selection system and demonstrating the exceptional plasmid transformation competence of NJ-SQYY.

**Fig. 2:**
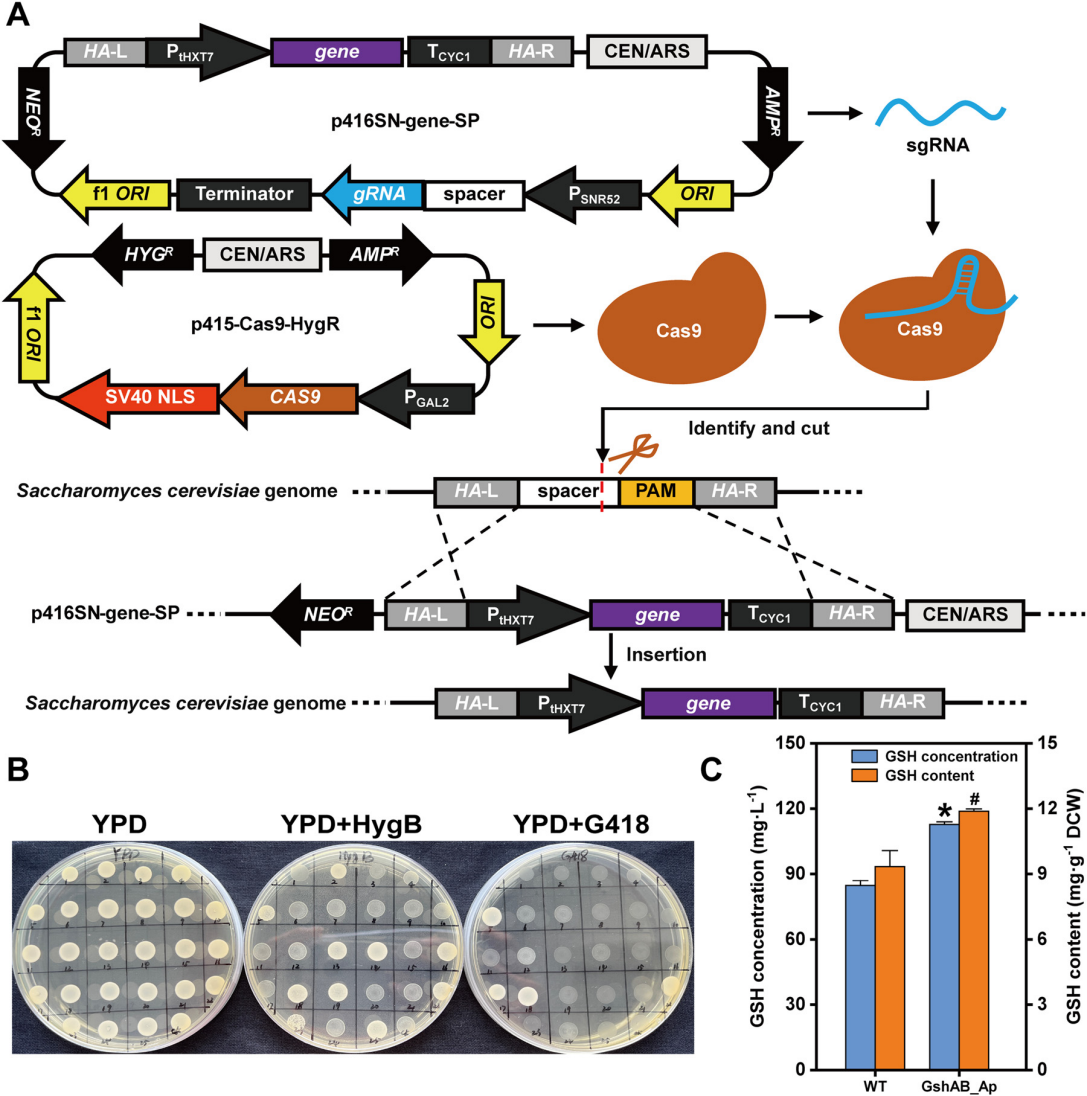
Working principle and results of the CRISPR/Cas9 system. A) Working principle of the two-plasmid CRISPR/Cas9 system. B) Validation results of the mutant strain NJ-SQYY/ *gshAB_Ap* losing plasmids. C) Comparison of glutathione levels between the wild-type NJ-SQYY and the mutant strain NJ-SQYY/ *gshAB_Ap*. Two strains were cultured in YPD medium for 48 h. Data represent the mean ± SD of triplicates.

To evaluate the genome-editing capacity of the dual-plasmid CRISPR/Cas9 system (comprising p415-Cas9-HygR and p416SN-gene-SP) (Fig. 2A), we integrated *gshAB_Ap*, which encodes a bifunctional glutathione synthase from *A. pleuropneumoniae*, into the CS6 locus (YNCG0042C) of the NJ-SQYY strain. As shown in Fig. S2B, all eight selected clones exhibited the expected fragment sizes, confirming the high knock-in efficiency of the CRISPR system for *S. cerevisiae*. Sequencing analysis of the PCR products from the representative mutant SQ1 verified precise integration without unintended mutations.

Following successful integration, plasmid curing was performed through antibiotic-free subculturing to eliminate potential metabolic burden during culture. Under non-selective conditions, yeast cells gradually lose nonessential plasmids by ceasing selective replication. Post-curing screening of the YPD plates containing hygromycin B or G418 identified 14 plasmid-free clones (Fig. 2B). Subsequent PCR verification of six randomly selected clones confirmed both plasmid elimination (absence of vector-specific bands) and stable genomic integration (persistent CS6 locus modification) (Fig. S2C)

Heterologous *gshAB_Ap* encodes GshAB_Ap, which exhibits reduced feedback inhibition by glutathione compared to the native yeast Gsh1 [11]. Culture analysis revealed a significant enhancement in GSH production in SQ1 derivatives compared to that in wild-type controls (Fig. 2C). Engineered strains showed a 33.14 % increased GSH yield (112.74 mg·L^-1^ vs 84.68 mg·L^-1^) and 27.28 % higher cellular GSH content (11.89 mg·g^-1^ DCW vs 9.34 mg·g^-1^ DCW), demonstrating functional expression of the integrated *gshAB_Ap* and its metabolic make a positive impact on GSH biosynthesis.

### 3.3 Combinatorial engineering of bifunctional glutathione synthase expression enhances GSH production

**Fig. 3:**
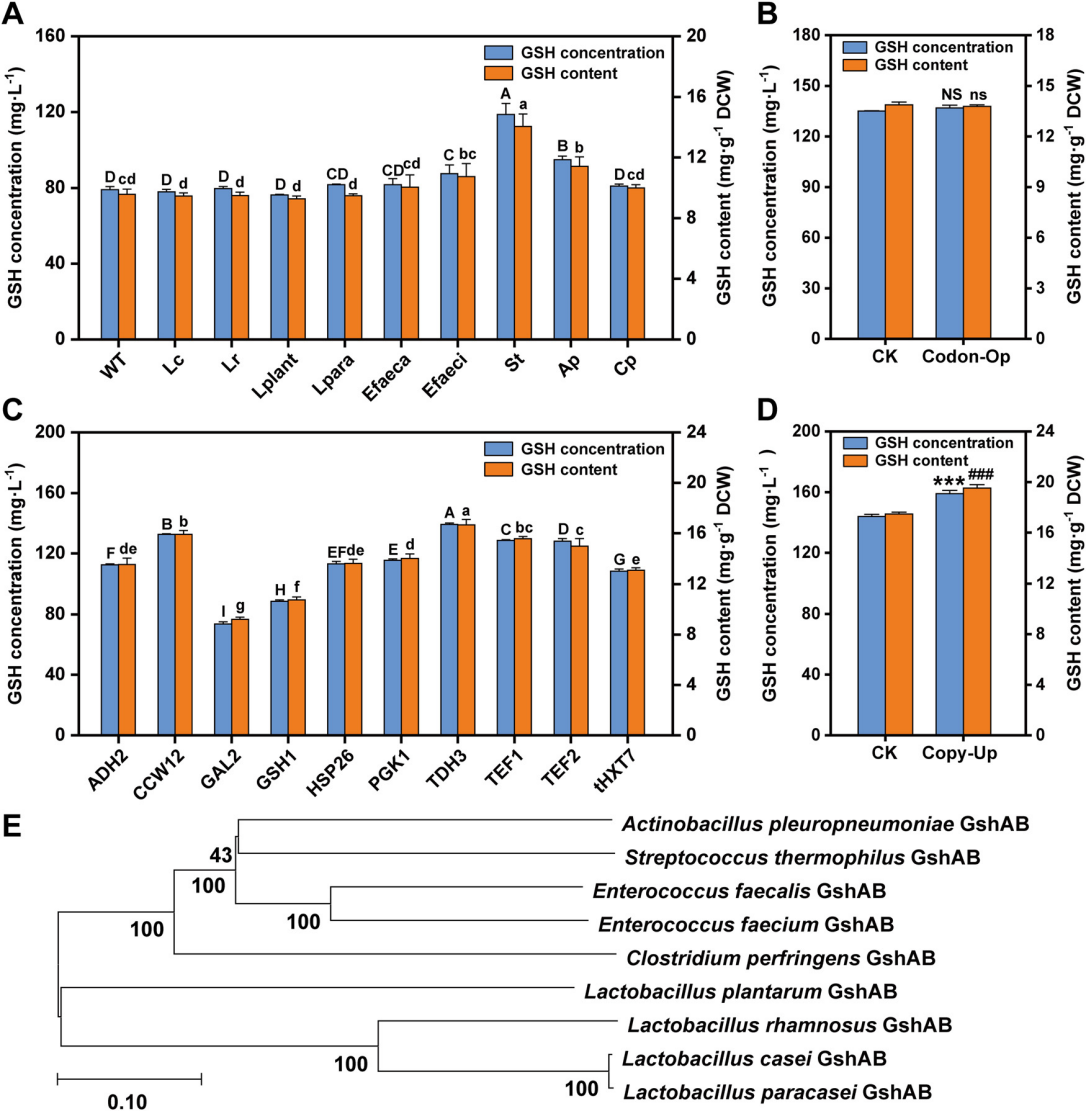
Comparison and optimization of the heterologous expression of GshAB for glutathione (GSH) synthesis in NJ-SQYY. A) GSH levels of strains heterologously expressing different GshAB in NJ-SQYY. B) GSH levels before and after codon optimization of *gshAB_St*. C) GSH levels after *gshAB_St* expression from different promoters. D) GSH levels before and after *gshAB_St* copy number increase. All strains were cultured in YPD medium for 48 h. Data represent the mean ± SD of triplicates. E) Phylogenetic tree of GshAB derived from different strains. The evolutionary history was inferred using the neighbor-joining method.

Although the heterologous expression of GshAB_Ap improved GSH biosynthesis in *S. cerevisiae* NJ-SQYY, this enhancement was limited. To identify superior orthologs, we searched for bacterial strains containing genes for the glutathione synthesis bifunctional enzyme in the NCBI database and then collected them to obtain *gshAB* genes from other sources. We systematically screened eight additional *gshAB* homologs from Gram-positive bacteria (*Lactobacillus casei, L. rhamnosus, L. plantarum, L. paracasei, Enterococcus faecium, Enterococcus faecalis, Streptococcus thermophilus*, and *Clostridium perfringens*). Comparative culture of engineered strains revealed that *S. thermophilus*-derived *gshAB_St* conferred the most significant improvement, with SQ8 (*gshAB_St*-integrated mutant) producing 118.65 mg·L^-1^ GSH (49.96 % increase vs. wild-type) and achieving 14.06 mg·g^-1^ DCW (46.99 % increase vs. wild-type) (Fig. 3A).

Phylogenetic analysis of the GshAB protein sequences (Fig. 3E) demonstrated high conservation among *Lactobacillus* spp. orthologs, none of which enhanced GSH production. In contrast, phylogenetically distinct enzymes from *S. thermophilus* and *A. pleuropneumoniae* showed superior functionality, suggesting that structure-activity relationships govern heterologous enzyme performance in yeast. In the available literature, it is evident that, despite the presence of the *gshAB* gene in the genomes of *Lactobacillus* spp., these organisms lack the ability to synthesize GSH. Specifically, GshAB in *L. plantarum* exhibited key amino acid differences compared to *S. thermophilus*, which led to a loss of enzymatic activity. Furthermore, in *L. casei, L. paracasei*, and *L. rhamnosus*, the GshAB enzyme is non-functional due to the absence of a 100-amino-acid sequence in the γ-glutamyl-cysteine synthetase (γ-GCS) domain. This deficiency prevents the synthesis of γ-glutamyl-cysteine (γ-GC), thereby inhibiting GSH production [23].

Although some researchers increased the expression of human cystatin C in *E. coli* from 10 % to 46 % by optimizing its codon to the preferred codon in the prokaryotic system [33], codon optimization of *gshAB_St* (SQ81) failed to enhance GSH production (Fig. 3B), likely because of the inherent compatibility of native codons with *S. cerevisiae* tRNA pools. Subsequent promoter engineering identified P_TDH3_ (P_GAP_) as the most effective regulatory element, driving SQ82 to produce 138.52 mg·L^-1^ GSH (a 30.27 % increase over P_tHXT7_ controls), with 16.68 mg·g^-1^ DCW (Fig. 3C). Because galactose was absent from the medium, the galactose-inducible promoter P_GAL2_, which was included as a control, drove basal expression only, as expected. Among the constitutive promoters tested, the endogenous P_GSH1_ exhibited the lowest activity.

Gene dosage escalation through tandem *gshAB_St* integration (SQ83) yielded diminishing returns (a 10.44 % increase in yield and an 11.72 % increase in content; Fig. 3D), suggesting cellular resource allocation thresholds and potential epigenetic silencing mechanisms. This observation aligns with previous reports on ceiling effects in metabolic engineering [40].

### 3.4 System-specific limitations in glutathione metabolic engineering: chaperonin-assisted folding and *YAP1*-mediated transcriptional activation fail to enhance production

**Fig. 4:**
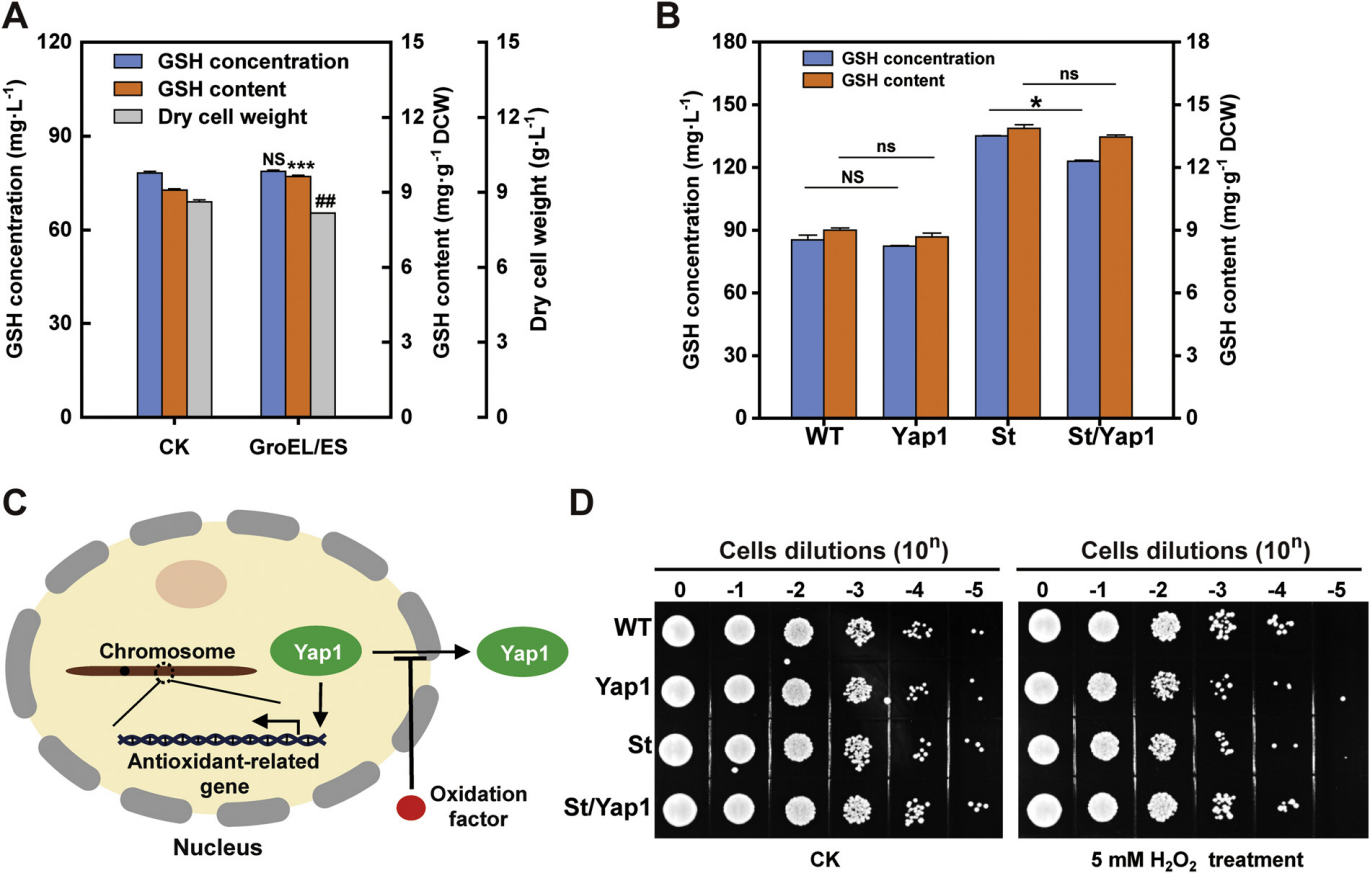
Results of the effect of GroEL/ES introduction and Yap1 overexpression on strains. A) Glutathione (GSH) levels in the NJ-SQYY/*gshAB_Ap* strain before and after the introduction of GroEL/ES. B) GSH levels before and after Yap1 overexpression in NJ-SQYY (WT) or NJ-SQYY/gshAB_St (St). All strains were cultured in YPD medium for 48 h. Data represent the mean ± SD of triplicates. C) Mechanistic diagram of Yap1 activation of antioxidant gene expression in response to stimulation with oxidative factors. D) Verification of the antioxidant capacity of Yap1-overexpressing strains.

The two-domain prokaryotic protein GshAB, which originates from prokaryotic sources, exhibits inefficient spontaneous folding and requires chaperone assistance to maintain its proper structural conformation. However, its heterologous expression in yeast (a eukaryotic host) may be incompatible with eukaryotic chaperone machinery, resulting in impaired functionality, as previously reported [11]. To address this limitation, we introduced the *E. coli* chaperonin GroEL/ES (encoded by the groSL operon) into the mutant strain NJ-SQYY/gshAB_Ap (SQ1), in line with previous reports, to ensure comparability and reproducibility [11]. Post-plasmid-curing culture assays yielded results that were partially consistent with expectations. Intracellular GSH levels increased significantly, likely because heterologous GroEL/ES expression partially assisted GshAB folding and enhanced its intracellular accumulation. However, this increase was accompanied by a marked reduction in biomass, which ultimately offset the increase in total GSH production (Fig. 4A). This pattern may reflect evolutionary differences in protein quality control systems between prokaryotes and eukaryotes, because the bacterial GroEL/ES chaperonin system is unlikely to function optimally within the redox environment and organelle organization in yeast. Previous studies have shown that the expression of *E. coli* GroEL/ES in *S. cerevisiae* can facilitate the folding of certain bacterial enzymes such as xylose isomerase and arabinose isomerase [36]. Nevertheless, heterologous chaperone expression often imposes substantial metabolic and resource burdens that compromise host growth without improving overall product yield [10,13]. Yeast cells exhibit an upper protein burden threshold beyond which growth is markedly impaired [4]. Collectively, these findings indicated that intracellular GSH accumulation increased in a predictable manner, whereas the concomitant reduction in biomass limited improvements in the overall GSH titer.

Yap1, a master transcriptional activator that governs the oxidative stress response in *S. cerevisiae*, regulates the genome-wide expression of antioxidant defense genes, including those involved in GSH biosynthesis [21]. To test whether *YAP1* overexpression could enhance endogenous GSH production via the upregulation of Gsh1 and Gsh2, we engineered *YAP1* integration into both the wild-type NJ-SQYY and the mutant strain NJ-SQYY/*gshAB_St* (SQ8). Post-curing culture analyses revealed paradoxical outcomes: *YAP1* overexpression failed to elevate GSH levels in either strain, with notable growth inhibition observed in the mutant (Fig. 4B).

This contrasts with established models in which the nuclear accumulation of Yap1 under oxidative stress drives antioxidant gene activation [27] (Fig. 4C). We hypothesized that the standard culture conditions maintained redox homeostasis, preventing sufficient oxidative stress to trigger the nuclear translocation of Yap1. Consequently, constitutive *YAP1* overexpression without proper activation signals may lead to dysregulated transcriptional activity or metabolic burden rather than pathway enhancement.

To further assess Yap1 function under oxidative stress and examine the relationship between cellular antioxidant capacity and GSH levels, exponentially growing cells were exposed to 5 mM hydrogen peroxide (H_2_O_2_) for 2 h, followed by viability assays. H_2_O_2_ treatment markedly reduced the number of colony-forming units in all strains (Fig. 4D). Under the conditions tested, *YAP1*-overexpressing strains may have exhibited a survival advantage. However, this apparent advantage did not translate into a significant enhancement of the overall antioxidant capacity, suggesting that antioxidant performance in the engineered strains is threshold-limited and that the simple transcriptional amplification of Yap1 targets is insufficient to counteract acute oxidative damage. An alternative explanation is that endogenous redox-buffering systems, including thioredoxin and peroxiredoxin networks, approach saturation, thereby constraining the phenotypic output of Yap1-dependent regulation under severe stress. Although the *gshAB_St*-expressing strain displayed higher GSH levels than the wild-type strain, it did not show stronger oxidative resilience, indicating that modest increases in GSH only make a minor contribution to whole-cell antioxidant capacity. In summary, *YAP1* overexpression did not markedly improve the global antioxidant phenotype, and low-amplitude changes in GSH content had a limited impact; consequently, we did not further pursue *YAP1*-based engineering in subsequent work.

### 3.5 Coordinated metabolic flux optimization through enzyme fusion and promoter engineering enhances GSH biosynthesis

**Fig. 5:**
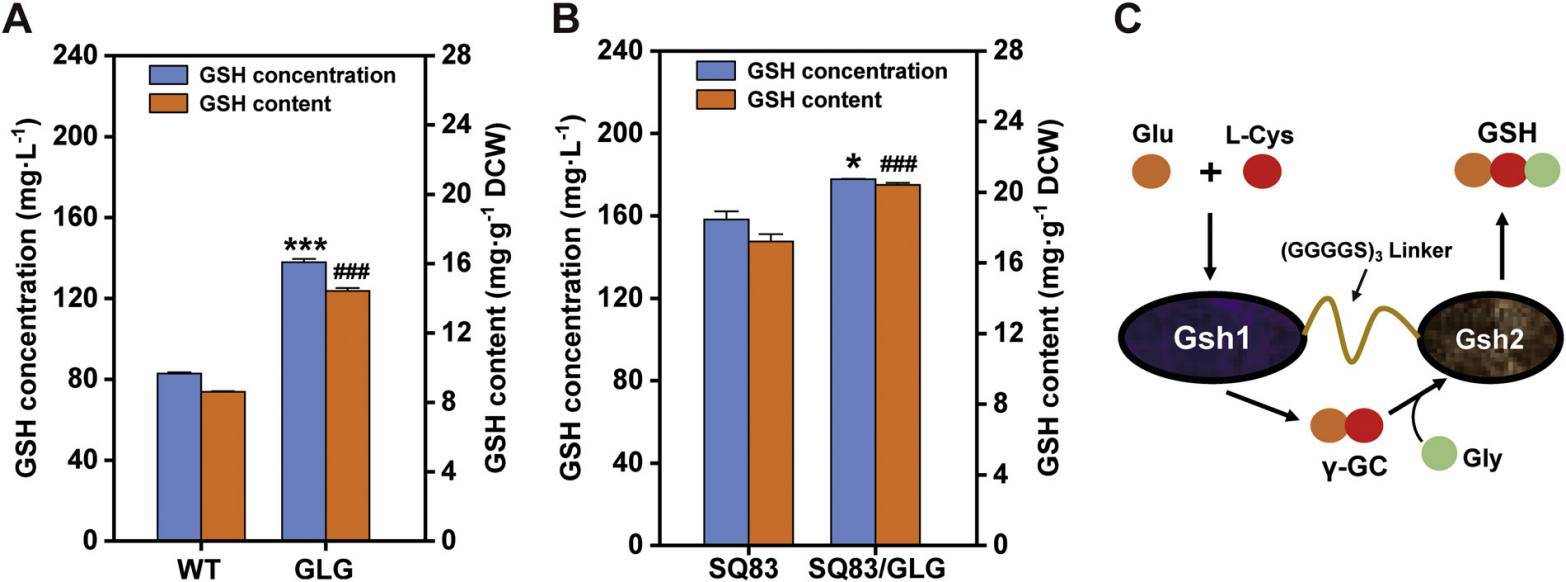
The Gsh1-Gsh2 fusion protein was expressed in the wild-type and engineered strain SQ83. A-B) Glutathione levels before and after Gsh1-Gsh2 fusion protein expression in the wild-type or engineered strain SQ83. All strains were cultured in YPD medium for 48 h. Data represent the mean ± SD of triplicates. C) Diagram of the structure of the Gsh1-Gsh2 fusion protein and its mechanism of action.

Strategic enzyme fusion has emerged as an effective approach for enhancing the catalytic efficiency of multi-enzyme biosynthetic systems. Researchers have demonstrated this principle by constructing a chimeric enzyme through the flexible linker-mediated fusion of three distinct cellulolytic enzymes that exhibit synergistic interactions, resulting in significantly elevated enzymatic activities compared to their individual counterparts [19]. To strengthen the endogenous GSH biosynthesis pathway in *S. cerevisiae*, which involves sequential catalytic steps mediated by Gsh1 and Gsh2, we implemented a fusion protein strategy. A flexible linker peptide [(Gly-Gly-Gly-Gly-Ser)₃] was employed to covalently connect Gsh1 and Gsh2, forming a bifunctional enzyme complex (designated *GLG*; Fig. 5C). Given that P_TDH3_ has already been employed for codon-optimized *gshAB_St* and endogenous *TDH3*, the chimeric cassette was expressed in P_CCW12_ to minimize the instability arising from promoter sequence redundancy [30]. P_CCW12_ exhibited activity second only to P_TDH3_ (Fig. 3C). The dual-function design achieved gene dosage amplification of both *GSH1* and *GSH2* while enabling substrate channeling to minimize intermediate diffusion losses, synchronize enzyme expression ratios, and enhance catalytic efficiency through spatial colocalization.

The *GLG* cassette was genomically integrated into the NJ-SQYY wild-type strain and engineered strain SQ83 (plasmid-cured derivative), yielding the corresponding strains, NJ-SQYY/*GLG* and SQ84. Culture analyses (Fig. 5A–5B) revealed that SQ84 achieved a GSH titer of 177.76 mg·L⁻¹ (20.41 mg·g⁻¹ DCW), representing 12.33 % and 18.64 % enhancements in production and cellular content, respectively, relative to the parental SQ83. Strikingly, NJ-SQYY/*GLG* exhibited more pronounced improvements of 66.63 % in titer and 67.40 % in content compared to the wild-type. This disparity suggests metabolic flux limitations in the engineered SQ83 background, in which precursor availability and energy cofactor supply may constrain pathway intensification. The observed substrate-level bottlenecks highlight the necessity of integrated metabolic engineering approaches that combine pathway optimization with redox cofactor balancing in advanced strain development.

### 3.6 Targeted overexpression of *CYS3* enhances GSH biosynthesis by alleviating the L-cystathionine bottleneck

**Fig. 6:**
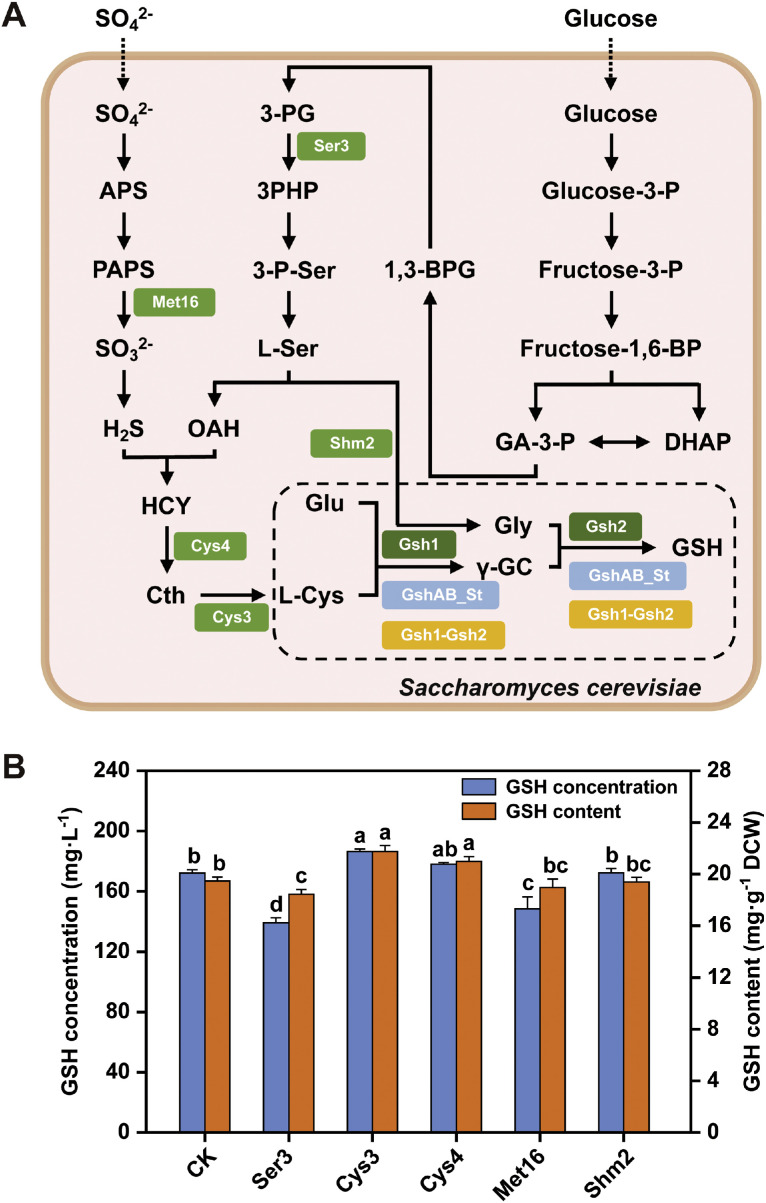
Glutathione (GSH) precursor synthesis pathway. A) Schematic representation of the GSH synthesis pathway in *S. cerevisiae* SQ84. APS, adenosine phosphosulfate; PAPS, 3-phosphoadenosine-5-phosphosulfate; HCY, homocysteine; Cth, cystathionine; OAH, O-acetyl-L-homoserine; 3-P-Ser, 3-phospho-L‑serine; 3PHP, 3-phoshydroxypyruvate; 3-PG, 3-phosphoglycerate; 1,3-BPG, 1,3-bisphosphoglycerate; GA-3-P, 3-phosphoglyceraldehyde; DHAP, dihydroxyacetone phosphate. B) GSH levels in SQ84 strains overexpressing genes of different precursor substance synthesis pathways. All strains were cultured in YPD medium for 48 h. Data represent the mean ± SD of triplicates.

To address potential metabolic bottlenecks in GSH precursor synthesis, we systematically analyzed the GSH biosynthetic pathway (Fig. 6A) and identified five rate-limiting enzymes based on literature evidence: Ser3 (3-phosphoglycerate dehydrogenase), Cys3 (cystathionine γ-lyase), Cys4 (cystathionine β-synthase), Met16 (adenylyl-sulfate reductase), and Shm2 (cytosolic serine hydroxymethyltransferase). The corresponding genes were individually amplified from the NJ-SQYY wild-type strain and genomically integrated into the SQ84 background using constitutive promoters, followed by plasmid curing and culture assays.

As shown in Fig. 6B, only the *CYS3*-overexpression strain SQ842 significantly enhanced both GSH titer (186.45 mg·L⁻¹) and cellular content (21.74 mg·g⁻¹ DCW), representing 4.89 % and 6.52 % increases compared to SQ84, respectively. This improvement likely stems from the alleviation of the L-cystathionine (Cth) accumulation bottleneck in the SQ84 metabolic network. Elevated Cys3 activity enhances the conversion of Cth to L-cysteine, thereby providing an increased substrate flux for GSH biosynthesis. Notably, the overexpression of the other four candidate genes (*SER3, CYS4, MET16*, and *SHM2*) failed to improve GSH production, suggesting that either redundant regulatory mechanisms or pathway crosstalk restricted their individual contributions. The strain-specific efficacy of *CYS3* overexpression underscores the context-dependent nature of metabolic engineering interventions, where precursor channeling and pathway compartmentalization critically affect the yields of target metabolites.

### 3.7 Strain-specific fermentation optimization reveals contrasting L-cysteine responsiveness for GSH overproduction

**Fig. 7:**
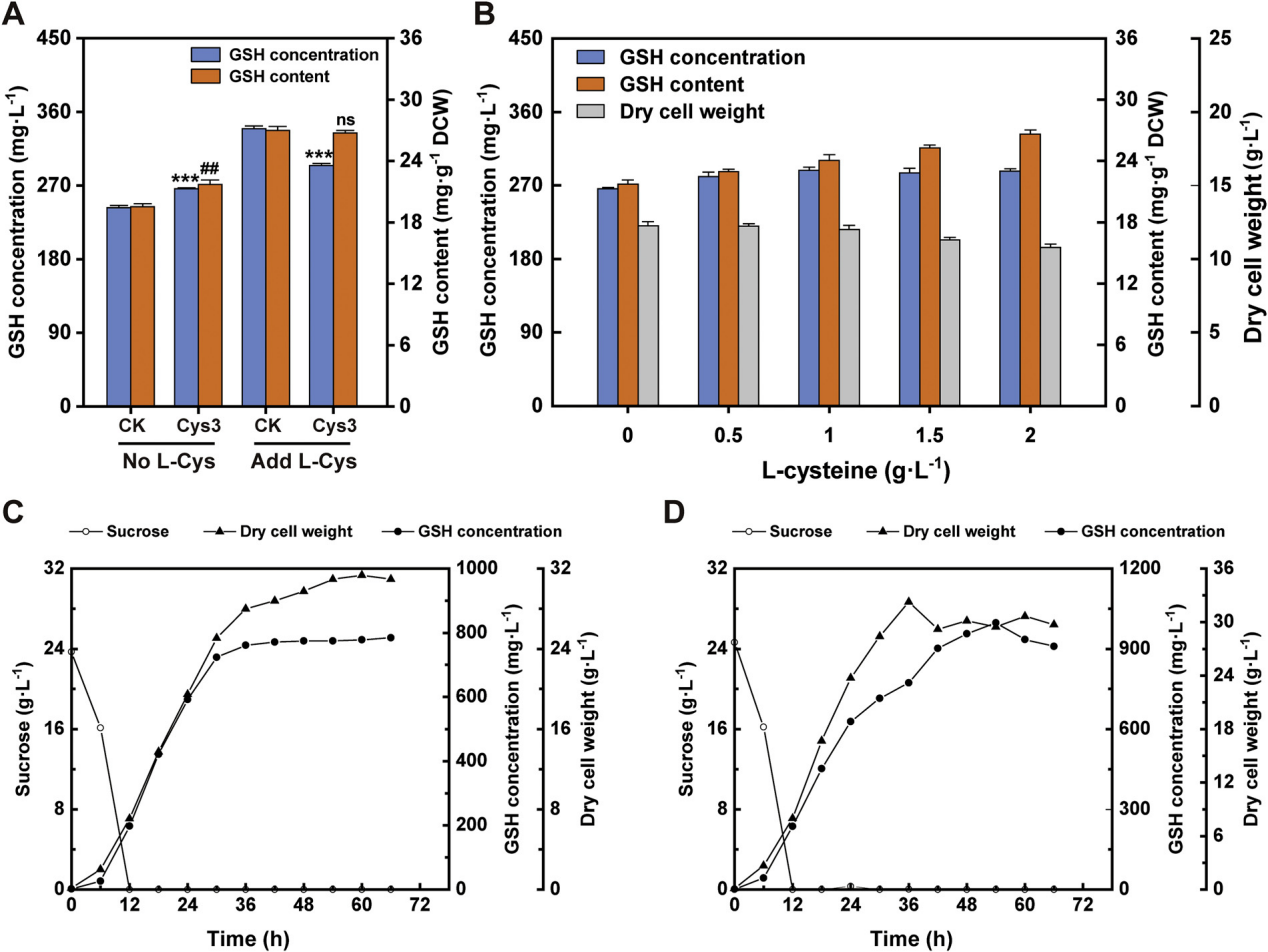
Fermentation of engineered yeast strains. A) Shaking flask fermentation of glutathione (GSH) levels in the presence or absence of L-cysteine in the Cys3 overexpression strain (SQ842) versus the control (SQ84). B) GSH levels of the strain overexpressing Cys3 (SQ842) under different concentrations of L-cysteine in shake flask fermentation conditions. L-cysteine was added at 24 h. The total fermentation time in the shaking flask was 48 h. C, D) Residual sugar, GSH concentrations, and dry cell weight of SQ842 and SQ84 strains fermented in a 5 L bioreactor; single run; no biological replicates; error bars not applicable.

To evaluate the fermentation capacity of the engineered strains SQ84 and SQ842 (SQ84/*CYS3*), we performed fed-batch fermentation using optimized media. Surprisingly, in the absence of exogenous L-cysteine, SQ842 achieved a GSH titer of 266.22 mg·L⁻¹ (21.71 mg·g⁻¹ DCW), surpassing that of SQ84 (Fig. 7A). However, supplementation with L-cysteine (2 g·L⁻¹) dramatically reversed this trend: SQ842 exhibited reduced GSH production (vs. the 339.30 mg·L⁻¹ in SQ84) despite comparable cellular content (26.98 mg·g⁻¹ DCW). This paradoxical response suggests that *CYS3* overexpression in SQ842 enhances endogenous L-cysteine synthesis to levels that exceed the cellular tolerance thresholds, leading to growth inhibition upon exogenous supplementation.

Dose-response assays (0–2 g·L⁻¹ L-cysteine; Fig. 7B) confirmed progressive biomass decline in SQ842 with increasing supplementation. While the maximal GSH titer (1 g·L⁻¹ L-cysteine) remained below the optimized yield of SQ84, this highlights the hypersensitivity of SQ842 to exogenous L-cysteine. Cys3 is induced by sulfur starvation and can be inhibited by the addition of cysteine to the growth medium [9]. Consequently, distinct fermentation strategies were implemented: SQ842 leveraged high-cell-density cultivation without supplementation, whereas SQ84 utilized L-cysteine feeding.

In 5-L bioreactor fed-batch fermentations (Fig. 7C), SQ842 reached peak biomass (approximately 30 g DCW·L⁻¹) at 42 h, and achieved a titer of 784.85 mg·L⁻¹ GSH (25.37 mg·g⁻¹ DCW) at 66 h finally. Rapid sucrose depletion provides opportunities to refine the feeding protocol. Conversely, SQ84 supplemented with 2 g·L⁻¹ L-cysteine (36 h) showed transient biomass fluctuations but produced 997.46 mg·L⁻¹ GSH (33.85 mg·g⁻¹ DCW) at 54 h (Fig. 7D), demonstrating phase-dependent precursor utilization efficiency.

## 4 Conclusions

This study implemented a four-stage engineering paradigm (chassis screening, exogenous pathway construction, expression optimization, and metabolic network rewiring) to achieve unprecedented GSH production in engineered *S. cerevisiae*. The final titer reached approximately 1 g·L^-1^, representing a 12.45-fold enhancement over the wild-type strain NJ-SQYY, with a shake flask yield of 339.3 mg·L^-1^.

The key methodological advances presented in this study include 1) cross-taxonomic enzyme mining for pathway construction, 2) a precursor-catalysis coordination framework balancing metabolic flux, and 3) cost-effective fermentation protocols. This integrated strategy establishes a scalable platform for microbial GSH biosynthesis and provides insights into engineering microbial cell factories that target high-value metabolites. These innovations address critical challenges in pathway modularity, host-prosthetic compatibility, and industrial scalability, demonstrating broad applicability in pharmaceutical and nutraceutical bioproduction.

Looking ahead, further gains in microbial GSH biosynthesis may be achieved by deploying sensor-guided dynamic control to decouple growth from production; reinforcing ATP/NADPH supply and sulfur assimilation while expanding cysteine/glycine precursor pools; engineering feedback-resistant, protease-stable variants of pathway enzymes and optimizing proteostasis; leveraging subcellular compartmentalization (e.g., mitochondria, peroxisomes) to increase catalytic efficiency; tuning transport/export and intracellular storage to mitigate product inhibition and enable in-situ recovery; and integrating multi-omics with ^13^C-MFA for model-guided, reactor-relevant optimization (e.g., fed-batch, pH/DO/redox control) alongside adaptive laboratory evolution.

## Data Availability Statement

Data will be made available on request.

## CRediT authorship contribution statement

**Zhiqi Hu:** Writing – original draft, Visualization, Validation, Software, Resources, Methodology, Investigation, Formal analysis. **Mengyuan Su:** Visualization, Validation, Resources, Methodology, Data curation. **Qibing Liu:** Validation, Software, Resources, Formal analysis, Data curation. **Ying Li:** Visualization, Software, Methodology, Data curation. **Yunxiang Liang:** Visualization, Resources, Conceptualization. **Shuangquan Li:** Project administration, Funding acquisition, Conceptualization. **Yingjun Li:** Writing – review & editing, Supervision, Project administration, Methodology, Funding acquisition, Formal analysis, Conceptualization.

## Declaration of Competing Interest

The authors declare the following financial interests/personal relationships which may be considered as potential competing interests:

Yingjun Li reports financial support was provided by Key Research and Development Project of Hubei Province. If there are other authors, they declare that they have no known competing financial interests or personal relationships that could have appeared to influence the work reported in this paper.

## Appendix Supplementary materials

Image, application 1
