# Supplementary material for: Systems metabolic engineering of glutathione biosynthesis in Saccharomyces cerevisiae: Pathway balancing coupled with enzyme screening for high-titer production
Source: Eng Microbiol. 2025 Sep 23;5(4):100243. doi: 10.1016/j.engmic.2025.100243 (PMC12967834; doi:10.1016/j.engmic.2025.100243)
Supplement: Supplementary file 1 [file mmc1.docx]

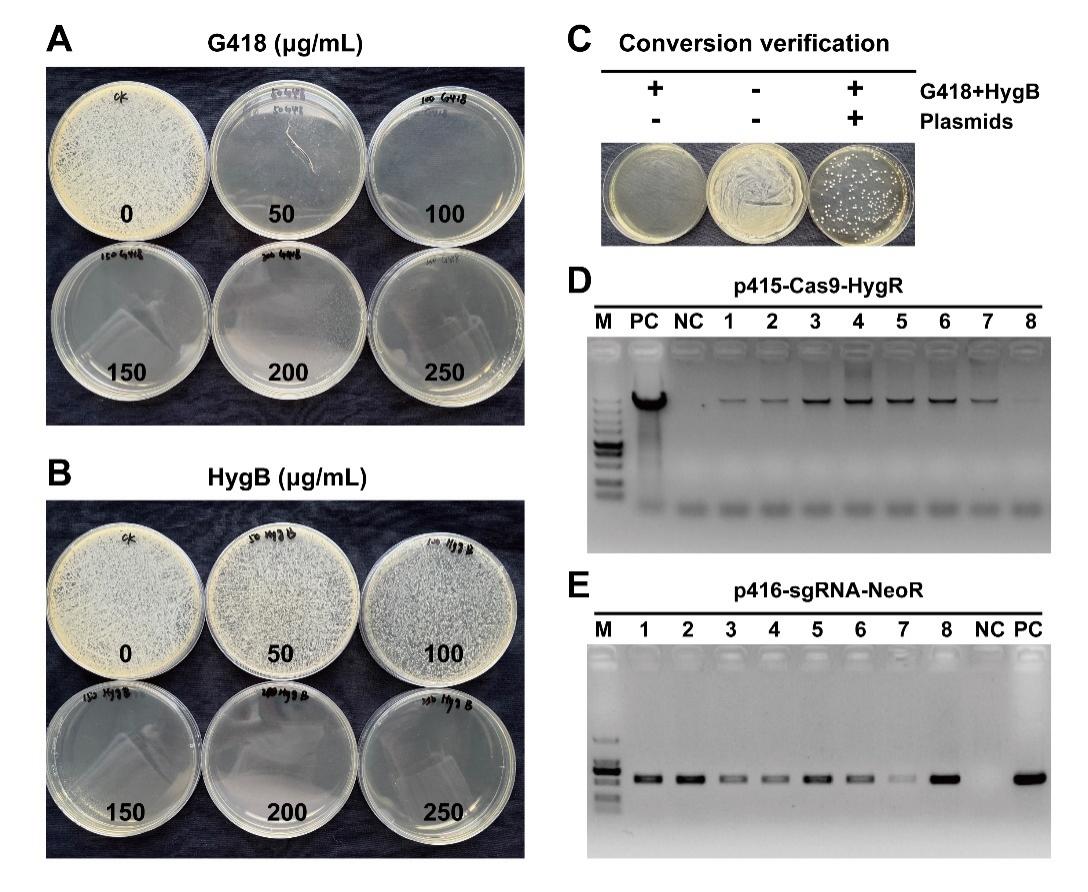


Supplementary Fig. S1 Validation of plasmid transformation ability of NJ-SQYY strain. A-B) The antibiotic resistance of NJ-SQYY to different concentrations of G418 or HygB. C) Plate results of the NJ-SQYY strain transformed with plasmids carrying antibiotic resistance genes. D-E) Results of agarose gel electrophoresis verified by plasmids transformants of strain NJ-SQYY. M, DNA maker; NC, the wild-type NJ-SQYY; PC, plasmid.


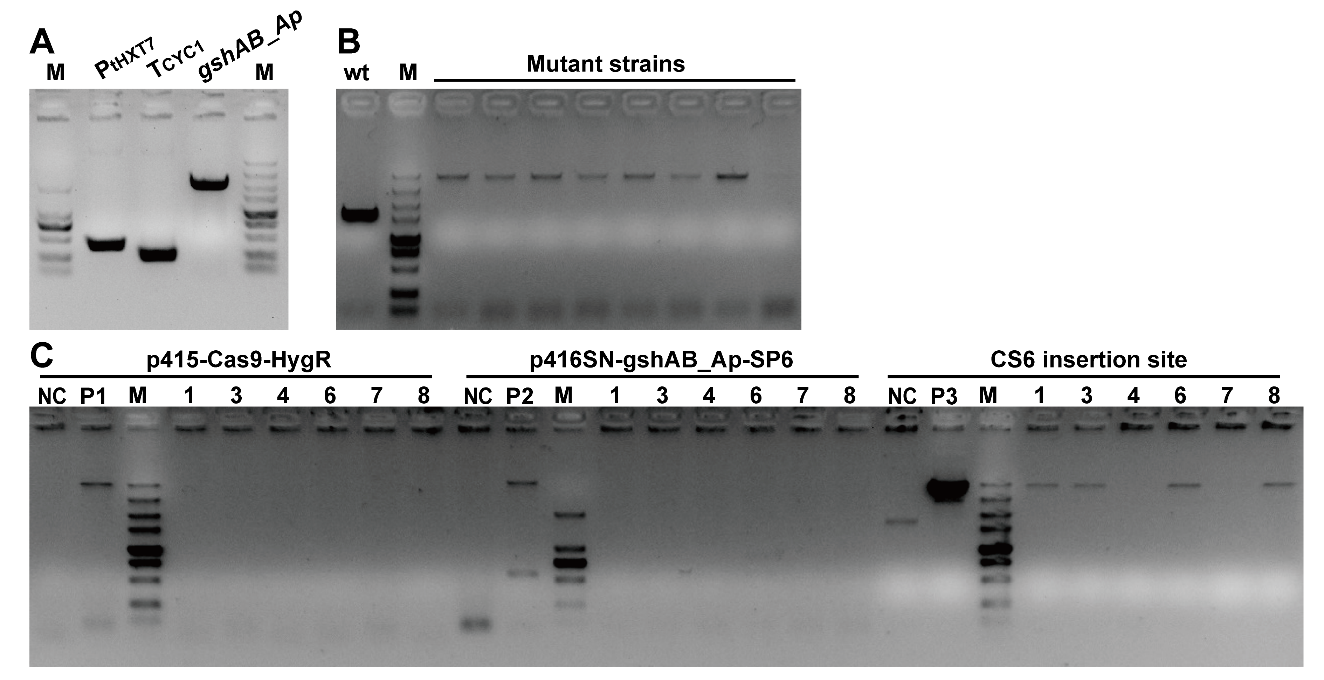


Supplementary Fig. S2 Validation results of *gshAB_Ap* knock-in NJ-SQYY. A) Results of agarose gel electrophoresis of DNA fragments of P_tHXT7_, T_CYC1_ and *gshAB_Ap*. B) Validation by agarose gel electrophoresis of gshAB_Ap knock-in NJ-SQYY. C) Validation results of the mutant strain NJ-SQYY/ *gshAB_Ap* losing plasmids. NC, the wild-type NJ-SQYY; P1, P2, plasmid; P3, the mutant strain NJ-SQYY/ *gshAB_Ap*.

Supplementary Table S1 Strains used in this study

| Strain | Relevant genotype/property | Source |
| --- | --- | --- |
| *E. coli*  *DH5a* | *F– endA1 glnV44 thi-1 recA1 relA1 gyrA96 deoR nupG purB20 φ80dlacZΔM15Δ(lacZYA-argF) U169, hsdR17(rK–mK+), λ–* | Lab storage |
| BY4741 | *Saccharomyces cerevisiae*, *MATa his3Δ1 leu2 met15Δ ura3-52* | Lab storage |
| NJ-SQYY | *Saccharomyces cerevisiae*, wild type | This study |
| NJ1447 | *Saccharomyces cerevisiae*, wild type | Lab storage |
| NJ-O | *Saccharomyces cerevisiae*, wild type | Lab storage |
| NJ-H | *Saccharomyces cerevisiae*, wild type | Lab storage |
| NJ-B | *Saccharomyces cerevisiae*, wild type | Lab storage |
| WA1339 | *Wickerhamomyces anomal*,*us*, wild type | This study |
| WA-J | *Wickerhamomyces anomalus*, wild type | This study |
| CJ-C | *Candida utilis*, wild type | This study |
| CJ-Z | *Candida utilis*, wild type | Lab storage |
| MK-1 | *Kluyveromyces marxianus*, wild type | Lab storage |
| MK-2 | *Kluyveromyces marxianus*, wild type | This study |
| SQ1 | *Saccharomyces cerevisiae* NJ-SQYY transformed with *gshABAp* | This study |
| SQ8 | *Saccharomyces cerevisiae* NJ-SQYY transformed with *gshABSt* | This study |
| SQ1g | *Saccharomyces cerevisiae* SQ1 transformed with *groSL* | This study |
| SQ8Y | *Saccharomyces cerevisiae* SQ8 transformed with *YAP1* | This study |
| SQ81 | *Saccharomyces cerevisiae* NJ-SQYY transformed with codon-optimized *gshABSt* | This study |
| SQ82 | *Saccharomyces cerevisiae* NJ-SQYY transformed with P_TDH3_-drived *gshABSt* | This study |
| SQ83 | *Saccharomyces cerevisiae* SQ82 transformed with codon-optimized *gshABSt* | This study |
| SQ84 | *Saccharomyces cerevisiae* SQ83 transformed with fusion expression protein gene *GSH1-GSH2* (*GLG*) | This study |
| SQ842 | *Saccharomyces cerevisiae* SQ83 transformed with *CYS3* | This study |

Supplementary Table S2 Yeast primary screening results

| **Serial number** | **Name** | **Number of strains** |
| --- | --- | --- |
| 1 | *Wickerhamiella pararμgosa* | 1 |
| 2 | *Starmera stellimalicola* | 1 |
| 3 | *Diutina catenμLata* | 1 |
| 4 | *Cyberlindnera jadinii* | 1 |
| 5 | *Saccharomycopsis schoenii* | 1 |
| 6 | *Galactomyces geotrichum* | 2 |
| 7 | *Trichosporon caseorum* | 3 |
| 8 | *Diutina rμgosa* | 1 |
| 9 | *Meyerozyma guilliermondii* | 2 |
| 10 | *Pichia fermentans* | 1 |
| 11 | *Pichia manshurica* | 2 |
| 12 | *Saturnispora mendoncae* | 1 |
| 13 | *Zygosaccharomyces bailii* | 1 |
| 14 | *Pichia kudriavzevii* | 3 |
| 15 | *Lodderomyces elongisporus* | 1 |
| 16 | *Candida glabrata* | 1 |
| 17 | *Candida parapsilosis* | 1 |
| 18 | *Wickerhamomyces anomalus* | 1 |
| 19 | *Papiliotrema flavescens* | 1 |
| 20 | *RhodotorμLa mucilaginosa* | 1 |
| 21 | *Saccharomyces cerevisiae* | 1 |
| 22 | *Kluyveromyces marxianus* | 1 |
| 23 | *Zygosaccharomyces parabailii* | 1 |
| 24 | *Zygosaccharomyces bailii* | 1 |

Supplementary Table S3 Plasmids used in this study

| **Plasmid** | **Description** | **Origin** |
| --- | --- | --- |
| p415-GalL-Cas9 | Skeleton of a plasmid expressing Cas9 protein | Lab storage |
| p416-SNR52-GTR | Skeleton of the plasmid expressing sgRNA | Lab storage |
| p415-Cas9-HygR | The screening marker for p415-GalL-Cas9 was changed to *HygR* | This study |
| p415-TEF2p-Cas9-HygR | The promoter of Cas9 was replaced by P_TEF2_ | This study |
| p416-sgRNA-NeoR | The screening marker for p416-SNR52-GTR was changed to *NeoR* | This study |
| p416SN-gshAB_Ap-SP6 | Editing plasmid for *gshAB_Ap* gene insertion | This study |
| p416SN-gshAB_Cp-SP6 | Editing plasmid for *gshAB_Cp* gene insertion | This study |
| p416SN-gshAB_St-SP6 | Editing plasmid for *gshAB_St* gene insertion | This study |
| p416SN-gshAB_Lr-SP6 | Editing plasmid for *gshAB_Lr* gene insertion | This study |
| p416SN-gshAB_Lc-SP6 | Editing plasmid for *gshAB_Lc* gene insertion | This study |
| p416SN-gshAB_Lpl-SP6 | Editing plasmid for *gshAB_Lpl* gene insertion | This study |
| p416SN-gshAB_Lpa-SP6 | Editing plasmid for *gshAB_Lpa* gene insertion | This study |
| p416SN-gshAB_Efa-SP6 | Editing plasmid for *gshAB_Efa* gene insertion | This study |
| p416SN-gshAB_Efi-SP6 | Editing plasmid for *gshAB_Efi* gene insertion | This study |
| p416SN-YAP1-SP1 | Editing plasmid for *YAP1* gene insertion | This study |
| p416SN-groSL-SP1 | Editing plasmid for *groSL* gene insertion | This study |
| p416SN-gshAB_St(op)-SP6 | Editing plasmid for *gshAB_St* (Codon-Op) gene insertion | This study |
| p416SN-gshAB_St(op)-SP6-ADH2p | Editing plasmid for P_ADH2_-*gshAB_St* (Codon-Op) gene insertion | This study |
| p416SN-gshAB_St(op)-SP6-CCW12p | Editing plasmid for P_CCW12_-*gshAB_St* (Codon-Op) gene insertion | This study |
| p416SN-gshAB_St(op)-SP6-GSH1p | Editing plasmid for P_GSH1_-*gshAB_St* (Codon-Op) gene insertion | This study |
| p416SN-gshAB_St(op)-SP6-HSP26p | Editing plasmid for P_HSP26_-*gshAB_St* (Codon-Op) gene insertion | This study |
| p416SN-gshAB_St(op)-SP6-PGK1p | Editing plasmid for P_PGK1_-*gshAB_St* (Codon-Op) gene insertion | This study |
| p416SN-gshAB_St(op)-SP6-TDH3p | Editing plasmid for P_TDH3_-*gshAB_St* (Codon-Op) gene insertion | This study |
| p416SN-gshAB_St(op)-SP6-TEF1p | Editing plasmid for P_TEF1_-*gshAB_St* (Codon-Op) gene insertion | This study |
| p416SN-gshAB_St(op)-SP6-TEF2p | Editing plasmid for P_TEF2_-*gshAB_St* (Codon-Op) gene insertion | This study |
| p416SN-GLG-SP3 | Editing plasmid for *GLG* gene insertion | This study |
| p416SN-CYS3-SP14 | Editing plasmid for *CYS3* gene insertion | This study |
| p416SN-CYS4-SP14 | Editing plasmid for *CYS4* gene insertion | This study |
| p416SN-SER3-SP14 | Editing plasmid for *SER3* gene insertion | This study |
| p416SN-MET16-SP14 | Editing plasmid for *MET16* gene insertion | This study |
| p416SN-SHM2-SP14 | Editing plasmid for *SHM2* gene insertion | This study |

Supplementary Table S4 Primers used in this study

| **Name** | **Sequence (5’ → 3’)** |
| --- | --- |
| Spacer (CS6)-F | AAACCTCCACAGATCGATACTTAT |
| Spacer (CS6)-R | TGCAATAAGTATCGATCTGTGGAG |
| Spacer (SPB1)-F | AAACTTGTACAATATTGAAAATAA |
| Spacer (SPB1)-R | TGCATTATTTTCAATATTGTACAA |
| Spacer (YPRCτ3)-F | TGCATAGTGGAACACATTCCAAGG |
| Spacer (YPRCτ3)-R | AAACCCTTGGAATGTGTTCCACTA |
| Spacer (YHRCδ14)-F | AAACCTGTATGTATGTGCCCTCGC |
| Spacer (YHRCδ14)-R | TGCAGCGAGGGCACATACATACAG |
| HR_HA (CS6)-F | TCTCCGGGAGCTGCATGTGTATGTTTCACGATGGAGAATGATAACACACTAAGTGG |
| HR_HA (CS6)-R | GATGACGGTGAAAACCTCTGATAAACGAATTTTGCGTACTTGTAACAAAATTACTTTTC |
| HR_HA (SPB1)-F | TCTCCGGGAGCTGCATGTGTATACACCTTCCGTGACACCGAAAAT |
| HR_HA (SPB1)-R | GATGACGGTGAAAACCTCTGGCCGGCACAATGAATGAAGT |
| HR_HA (YPRCτ3)-F | TCCGGGAGCTGCATGTGTATATATTCTAAAATATTGATAATTAATGGCAAAAAGGCAGT |
| HR_HA (YPRCτ3)-R | GATGACGGTGAAAACCTCTGTGGTCGTTTTTGTGCAGCATATTGT |
| HR_HA (YHRCδ14)-F | TCTCCGGGAGCTGCATGTGTGTAGTAGTTCCATGAAGTCTAATGACACT |
| HR_HA (YHRCδ14)-R | GATGACGGTGAAAACCTCTGGAAATTTTACAGCAACAGAAAGCACAGT |
| CS6-F | cgaaagctctaccattgagccaccgcttc |
| CS6-R | ctgcccaatattcctttttcaatgggtttctaggcat |
| SPB1-F | gagagttcaagtgatgactccgatttcga |
| PBN1-R | gcatacaatcactcgacgactacgac |
| YPRCτ3-F | GGATACGTCAGTATGACAATACTTCATCCTAAACGT |
| YPRCτ3-R | gtattgtggtgttcttttcgatattctcagataccgt |
| YHRCδ14-F | gcaattaatgaaccatcgccaatttttgct |
| YHRCδ14-R | agctcatttccaaaacttcagttcgcct |
| BsaI-gshAB_ Ap-F | gctaGGTCTCgaaaaATGAAATTACAACAACTAATTAAAACTCACCAAC |
| BsaI-gshAB_Ap-R | gatcGGTCTCgATGATTAAGGCAGTTCTGGGAACAG |
| HR-gshAB_Cp-F | taattttaatcaaaaaatggtgaatttagataaaggtttattaaaaattataaaagatg |
| HR-gshAB_Cp-R | ACATAACTAATTACATGAttaattaaacaataaatctaatatcttttcaccagcttttc |
| HR-gshAB_Efaeca-F | tttttaattttaatcaaaaaatgaattatagagaattaatgcaaaagaaaaatgttcg |
| HR-gshAB_Efaeca-R | TGACATAACTAATTACATGAttattgaaccacttctgggtataaaagttttaaaac |
| HR-gshAB_Efaeci-F | tttttaattttaatcaaaaaatgatgaattttaagcaattattattgcatgtc |
| HR-gshAB_Efaeci-R | GACATAACTAATTACATGAttaaaatacttcaggatataacaatttcaatacattcatc |
| HR-gshAB_Lc-F | tttttaattttaatcaaaaaatgttgaatcattattggcaactgatcc |
| HR-gshAB_Lc-R | TGACATAACTAATTACATGAttatttttcgctaaacagatacgtgagtaaag |
| HR-gshAB_Lpara-F | tttttaattttaatcaaaaaatgttgaatcattattggcaactgatcc |
| HR-gshAB_Lpara-R | TGACATAACTAATTACATGAttatttttcgctaaacagatacgtgagtaaag |
| HR-gshAB_Lplant-F | tttttaattttaatcaaaaaatggaattagatgccgttggtaag |
| HR-gshAB_Lplant-R | TGACATAACTAATTACATGAttaatcttcatttttaaacaatgcatccaacaac |
| HR-gshAB_Lr-F | tttttaattttaatcaaaaaatgaatcattattggcaactgattcg |
| HR-gshAB_Lr-R | TGACATAACTAATTACATGAttatctcacagcaaacagccagt |
| HR-gshAB_St-F | tttttaattttaatcaaaaaatgacattaaaccaacttcttcaaaaactg |
| HR-gshAB_St-R | TGACATAACTAATTACATGAttaagtttgaccagccactatttctg |
| tHXT7p-F | tcgtaggaacaatttcgggc |
| BbsI_tHXT7p-R | catgagcGTCTTccggcGAAGACgctttttgattaaaattaaaaaaactttttgtttttgtgttta |
| BbsI_CYC1t-F | gcGTCTTCgccgGAAGACgcTCATGTAATTAGTTATGTCACGCTTAC |
| CYC1t-R | GCAAATTAAAGCCTTCGAGC |
| ADH2p-F | GAAGTTTTTTTACCCCTCTCtatcttaactgatagtttgatcaaaggggc |
| ADH2p-R | AACAATTGGTTCAAAGTCATtgtgtattacgatatagttaatagttgatagttgat |
| CCW12p-F | GAAGTTTTTTTACCCCTCTCGGATACTTCATGCTATTTATAGACGC |
| CCW12p-R | AACAATTGGTTCAAAGTCATTATTGATATAGTGTTTAAGCGAATGACAG |
| GAL2p-F | GAAGTTTTTTTACCCCTCTCGCAACTACTTTGCATCAAACTCCAAT |
| GAL2p-R | AACAATTGGTTCAAAGTCATTATGAAAGAATTATTTTTTTTATTATGTTAATCTTGTGT |
| GSH1p-F | GAAGTTTTTTTACCCCTCTCcacacacgtattcttgtgcacac |
| GSH1p-R | AACAATTGGTTCAAAGTCATtttattcttctatatgtatattttcgatactctaaacc |
| HSP26p-F | GAAGTTTTTTTACCCCTCTCtagcttacagtaagccacaattctctt |
| HSP26p-R | AACAATTGGTTCAAAGTCATgttaatttgtttagtttgtttgtttgctt |
| PGK1p-F | GAAGTTTTTTTACCCCTCTCgagtgaggaactatcgcatacct |
| PGK1p-R | AACAATTGGTTCAAAGTCATtgttttatatttgttgtaaaaagtagataattacttcct |
| TDH3p-F | GAAGTTTTTTTACCCCTCTCtcattatcaatactgccatttcaaagaat |
| TDH3p-R | AACAATTGGTTCAAAGTCATtttgtttgtttatgtgtgtttattcgaaact |
| TEF1p-F | GAAGTTTTTTTACCCCTCTCccacacaccatagcttcaaaat |
| TEF1p-R | AACAATTGGTTCAAAGTCATtttgtaattaaaacttagattagattgctatgct |
| TEF2p-F | GAAGTTTTTTTACCCCTCTCtaacatcaatcatgcggttgct |
| TEF2p-R | AACAATTGGTTCAAAGTCATgtttagttaattatagttcgttgaccgt |
| HR-YAP1-F | taattttaatcaaaaaatgagtgtgtctaccgccaag |
| HR-YAP1-R | ACATAACTAATTACATGAttagttcatatgcttattcaaagctaattgaacgt |
| groS-KL-F | atgaatattcgtccattgcatgatcgcgtgatcg |
| groS-KL-R | ttacgcttcaacaattgccagaatgtcgctttc |
| groL-KL-F | atggcagctaaagacgtaaaattcggtaacgacg |
| groL-KL-R | ttacatcatgccgcccatgccaccca |
| groSL-KL-F | gctaGGTCTCgaaaaatgaatattcgtccattgcatgatcgcgtgatcg |
| groSL-KL-R | gatcGGTCTCgATGAttacatcatgccgcccatgccac |
| CCW12-GSH1-F | GCTTAAACACTATATCAATAatgggactcttagctttgggcac |
| GSH1-R | acatttgctttctattgaaggcttatttttttttacatattctgcaat |
| G-L2-GSH1-R | gcttcctcctcctccagaaccaccaccaccacatttg |
| G-L1-GSH2-F | cttcaatagaaagcaaatgtggtggtggtggttctgcacactatccaccttccaaggat |
| CYC1t-GSH2-R | TGACATAACTAATTACATGActagtaaagaataatactgtccaaacatccgaatcct |
| HR-CYS3-R | TGACATAACTAATTACATGAttagttggtggcttgtttcaag |
| HR-CYS4-F | tttttaattttaatcaaaaaATGACTAAATCTGAGCAGCAAG |
| HR-CYS4-R | TGACATAACTAATTACATGATTATGCTAAGTAGCTCAGTAAATCCAT |
| HR-MET16-F | tttttaattttaatcaaaaaATGAAGACCTATCATTTGAATAATGATATAATTGTC |
| HR-MET16-R | TGACATAACTAATTACATGACTAGGCATCTTGCTTTAAAAATTGC |
| HR-SER3-F | tttttaattttaatcaaaaaATGACAAGCATTGACATTAACAACTT |
| HR-SER3-R | TGACATAACTAATTACATGATTAATATAGCAATCTAATTGAGATCTTAGCAGAGGT |
| HR-SHM2-F | tttttaattttaatcaaaaaATGCCTTACACTCTATCCGAC |
| HR-SHM2-R | TGACATAACTAATTACATGATTACACAGCCAATGGGTATTCG |
| HP-p416-F | cagaggttttcaccgtcatcaccgaaacgc |
| HP-p416-R | ACACATGCAGCTCCCGGAGACGGTC |

**Supplementary Table S5** Exogenous gshAB gene sequences

| >gshAB_Ap | ATGAAATTACAACAACTAATTAAAACTCATCACCTTGGTTTACTATTTCAACAAGGTAAATTTGGCATCGAAAAAGAAAGCCAACGCATTGATAATAAAGGGAATATTGTTACTACCGCCCATCCTAGCGTTTTTGGTAACCGCAGTTATCATCCGTATATTCAAACCGATTTTGCAGAAAGTCAGTTAGAACTTATCACACCACCAAACGATACATTGGAAGACACATATCGTTGGCTATCGGCTATTCACGAGGTAACGTTACGTTCGTTGCCCGATGATGAATATATTTTCCCATTCAGCATGCCTGCCGGTTTACCGCCGGAATTCGAAATCAAAGAAGCACAATTAGATAACGAATGGGACGTGAAATATCGTGAACACCTTTCTGCCATTTATGGCAAATACAAGCAAATGGTGAGCGGTATTCACTATAATTTCCAAATTTCCGAAGAATTTGTCGAAAGCACATTTGCATTACAAACGGAATACCGCGATAAAATTGCGTTCCGCAATGCGCTATATATGAAATTAGCCAATAACTTTTTACGTTATCAATGGATTTTAGTTTATTTGCTTGCCGCAACCCCAACTGTAGAAGCGCAATATTTCGGTAAAAACTCACCGCTTGCAGAAGGGCAATTAGTACGTAGTTTACGTTCCGGCCCTTATGGTTATGTAAATGCGCCACATATTGTGATCAACCACGACAGCTTGCAACAATATGTCGAGTCGCTAGAACATTTTGTAGCAACCGGCGATTTGTTGGCAGAAAAAGAATTCTATTCAAACGTTCGTTTACGTGGTGCGAAAAAAGCACGCGAATTGCTTGAGAAAGGGGTTAAATATGCGGAATTCCGTTTATTTGATCTTAATCCGTTCTCGCCTTACGGTATCGAGCTTGCAGACGCGAAATTTATTCATCTGTTCTTACTTGCGATGTTGTGGATGGATGAAACAAGCGGTCAAAGAGAAGTCGAAATCGGCACACAAAAACTATACCAAGTTGCCCTTGAAGATCCTCGTTCGCACACTGCGTTCCAAGCAGAGGGTGAGGCGATCCTTAACCTGATGCTGGCAATGCTGGACGATCTTTCTGTACCACAAAACGAGAAAGATTTATTACAACAAAAACTGGCACAATTTGCCGATCCTAGTCAAACGGTAAACGGTCGTTTATTAGCCGCAGTTGAACAAGCCGGCAGCTATAAAGCTCTCGGTGCACAACTTGCTCAACAATATAAAGCGCAAGCATTCGAGCGTTTTTATGCGATTTCCGCTTTCGATAATATGGAGCTTTCTACACAGGCTTTGTTATTTGATGCGATCCAACAAGGCTTACAGATCGAATTGCTTGATGAAAACGATCAGTTCCTCGCACTCAAATTCGGCGATCATCTCGAATATGTGAAAAACGGCAATATGACCAGCCACGATCAGTATATTTCGCCATTAATTATGGAAAACAAAGTCGTAACCAAAAAAGTGTTGGCGAAAGCCGGTTTTAATGTGCCGAAAAGTGTTGAATTTACCTCTGTAGAACAAGCGGTGGCACACTATCCGTTATTTGAAGGTAAAGCGGTGGTAATTAAGCCGAAATCAACTAATTACGGCTTAGGTATTACATTTTTCCAGCAAGGCGTGACGGATAAAGCCGACTTTGCCAAAGCGATTGAAATTGCGTTCCGTGAAGATAAAGAAGTGATGGTGGAAGACTATTTAGTCGGCACCGAATACCGTTTCTTTGTGTTAGGCGATGAAACACTGGCGGTATTGTTACGTGTGCCGGCAAATGTGAAAGGTGACTGCATACATACAGTGCGTGAATTGGTGGAAGCGAAAAACAGTGATCCGCTACGAGGTGACGGCTCTCGTTCACCATTGAAGAAAATCGCCCTCGGTGACATTGAATTGCTTCAGCTTAAAGAGCAAGGTTTAACGCCTGATTCGATTCCGGCTGACGGGCAAATCGTACAATTACGTGCCAACTCTAATATTAGTACCGGCGGCGATTCAATCGATATGACCGATCAAATGCATGACAGTTATAAACAATTAGCGGTCGGTATTGCCAAAGAGATGGGGGCAAAAGTCTGCGGTGTGGATTTAATCATTCCGGATTTAACCAAAGCCGCCGAGCCGTCTCTGCGTTCATGGGGTGTGATTGAAGCAAACTTTAATCCGATGATGATGATGCATATTTTCCCTTACCAAGGAAAATCTCGCCGCTTAACCAAAGCCGTGTTAAAAATGCTGTTCCCAGAACTGCCTTAA |
| --- | --- |
| >gshAB_Cp | ATGGTGAATTTAGATAAAGGTTTATTAAAAATTATAAAAGATGAAAGTTTAGAAGATTACTTTATAAAGGCTAATTTTGGTTTAGAAAAAGAAAATGTTAGGGTTACAGAAAGCGGAAACTTAGCTTTAACACCTCATCCAAAGGCCTTTGGAGATAGGGAAAAGAATGCATATATAAAAACAGATTTTTCTGAGAGTCAGTTAGAAATGGTAACACCTGTCTGTAATACTTTAGAGGAAGTTTATAGTTTTATATGTAATTTAAATAAGGTTGTATCTTTAGAGATTATGAAAAATGGAGAATTTTTATGGCCACAGAGTAATCCTCCTATATTACCAAGGGAAGAGGAGATTCCAATTGCTAAGCTTTCTAATAGAGAAGATGAATTATATAGAGAAAATCTTAGTTATAAATACGGCAAAAAGAAACAAGTAATAAGTGGAATACATTATAATTTTTCATTTAAAGAGGAATTTATTAAGTTACTTTATAAAGAGCTTAAAGTGGAAAAGGATTTTAGAGAATTTAAAGATGATATCTACCTTAGAATGGCTAGAAACTTTCAAAAATATCATTGGTTATTAATATACTTAACTGGAGCAAGTCCTGTTTTCCATGAAAGTTATATAGATGAGATTAAAAAAGATGGTGAAATTTTAGGAGAGGATTCTTATTATATAAAAGATGATACTTCTTTAAGAAATAGTTCTTATGGATATAAAAATAAAAAGGACTATTATGTTTCATATAACAGTATAGGAGAATATGCTAGTGACATAAAGAATTTAGTTAAGGATAAGGAAATACAAAGTATAAAAGAATATTATAACCCTATAAGGTTAAAATCACTAGGAAGCGAAGACATGCTTGAAAGCTTACTTCACAAGGGGATTGATTACTTAGAGGTAAGACTTTTAGACTTAGATCCTTTAAGTATTCAAGGAGTGAGTAAAGAAACACTTTATCTATTGCACTTATTTATGATTTATACTTTATTAAAAGAGAACAAGGAAATAACATATAAAGATCAAGAAGAATTTTTCAAAAATCATGATATGGTTGCCTTAAAGGGCAGAAATGAAGACGCTGTTATACATGAAAATGGAGTTCCTGTTTTATTAAAAGACAAAGGAAGAGAAATACTAAGTGAAATGGATGAAATTGTAGAGATCTTATTTTCAAATAATGAGGAATTTAAAAATGTTATCAAAAGAGCATTAGAAAAAATTAATAATCCTCATGATATAATTTCAGAGAAACTTATTAAGTATATAAAAGAAGAGGGATATATTAATTTCCATATGAGATTAGCTAAGGAGTACTTAAATAACTTTAAAAATAAAGAATTTAATTTAGTTGGTTATGAAGATTTAGAATTATCAACACAAATATTAATATTAGATGCTATTAAAAGAGGAATAGAGTTTAATATGATGGATAGATTAGAGAACTTTATTTCTCTTAGTGATGGAGAAAAGGTTGAATATGTTAAGCAAGCAACAAAAACTTCTAAGGATTCATATATAACTGCCTTAATAATGGAAAATAAATTAGTTACTAAGGATATTTTAAGGGAAAATAATATAAGGGTTCCTAAGGGGAAAGATTATGACAATATAGATGAGGCAAAGAAAGATTTTAGATTATTTAAGGATGAGAAAATAGTTATAAAACCTAAGTCTACTAATTTTGGTTTAGGAATTAGTATATTTCCTGGAGAATATTCAAGGGAAGACTATGATAAAGCTGTTGAAATAGCTTTTAGAGAGGATAGTTCAATCCTTATAGAAGAGTTTATGACAGGAAAGGAATATAGATTTCTTGTAATAGGGGAAGAGGTAGTAGGAATACTTCATAGAGAACCTGCCAATGTAATTGGTAATGGAGAAAGTACCATAGAAGAACTTGTTTTTGAAAAGAATAAAGATCCATTAAGAGGAAAGGGATATAAGACTCCTTTAGAAAAAATAAAATTAGGAGAGATAGAGGAAATGTTTTTGAAAAATCAAGGACTAAGCTTTAAGTCTATCCCTAAAAATGGAGAAAAAATCTATTTAAGAGAAAACTCTAATATAAGTACAGGAGGAGATAGTATAGACTTTACTGACAAAATACATCCTAGTTATAAAGAGGTAGCATTAAAGTCTGCTAAGGCTGTTAAAGCCCTTATATGCGGAGTAGATATGGTAATAGATAATATAGAGGAAGAGGCAAAGGAAAAAAATCATGGCATAATAGAATTGAATTTTAACCCAGCAATACATATTCATTGTTTCCCTTATAAAGGAGAGAATAGAAAAGCTGGTGAAAAGATATTAGATTTATTGTTTAATTAA |
| >gshAB_Efaeca | ATGAATTATAGAGAATTAATGCAAAAGAAAAATGTTCGTCCTTACGTATTGATGGCTCGTTTTGGTTTAGAAAAAGAAAACCAACGTAGTACACGAGAAGGGCTTTTAGCGACAACTGATCATCCCACGGTTTTTGGTAACCGTTCTTATCATCCATATATTCAAACAGATTTTAGTGAAACACAATTAGAACTAATCACGCCTGTAGCAAATAGCGGCACAGAAATGCTTCGTTTTTTAGATGCCATTCACGATGTGGCTCGTCGTTCGATTCCAGAAGATGAAATGCTGTGGCCATTAAGTATGCCGCCACAATTACCAACAAAAGATGAAGAGATTAAAATTGCTAAATTAGATCAATATGATGCAGTGTTATATCGTCGTTATTTGGCAAAAGAGTATGGCAAACGAAAACAAATGGTCAGCGGAATTCATTTTAATTTTGAATATGACCAAGCCCTGATTCAGCAATTATATGATGAACAATCCGAAGTGACAGATTGCAAACAATTTAAAACGAAAGTGTACATGAAAGTTGCCCGTAACTTTTTACGTTATCGTTGGTTAATTACGTATCTTTTTGGGGCTTCGCCAGTTAGTGAAGACGGCTACTTTAGAGTCTATGACGACCAACCGCAAGAACCCATTCGCAGTATTCGGAATAGTACGTATGGCTACAGAAATCATGACAATGTGAAAGTATCGTATGCCTCATTGGAACGCTATTTAGAAGATATTCATCGCATGGTGGAAAATGGTTTACTTTCTGAAGAAAAAGAATTTTATGCGCCTGTGCGCTTACGTGGTGGGAAACAAATGTCTGATCTGCCTAAAACAGGTATTCGCTATATCGAGTTGCGTAATTTAGACTTAAATCCTTTTTCACGTTTAGGCATTGTGGAAGATACTGTGGATTTCTTACATTATTTCATGTTGTATTTATTGTGGACAGATGAAAAAGAAGAAGCGGATGAATGGGTAAAAACTGGCGATATTTTAAATGAACAAGTGGCTCTTGGTCATCCTCATGAAACGATTAAGTTAATTGCAGAAGGCGATCGGATTTTTTCAGAAATGATTGATATGTTAGATGCTCTAGGCATTCGTAAAGGCAAAGAAGTTGTCGGTAAGTATTATCAACAACTGCGGAATCCACAAGACACCGTTTCTGGCAAAATGTGGACGATTATTCAAGAAAACTCCAACAGTGAACTGGGAAATATTTTTGGAAACCAATATCAAAGTATGGCCTTTGAACGCCCTTATCAATTAGCTGGTTTCCGTGAGATGGAATTATCCACACAAATTTTCTTGTTTGATGCGATTCAAAAAGGTTTGGAAATCGAAATTTTAGATGAACAAGAGCAATTTTTGAAACTGCAACATGGCGAGCACATTGAATACGTCAAAAATGCCAACATGACTAGCAAAGATAACTACGTGGTACCATTGATTATGGAAAACAAAACCGTGACAAAGAAAATTTTGTCTGCAGCAGGGTTCCATGTGCCTGGCGGTGAAGAATTTTCATCTTTTATTGAGGCACAAGAAGCACATTTACGCTACGCCAATAAAGCGTTTGTCGTGAAACCAAAATCAACGAATTACGGTTTAGGAATTACCATTTTTAAAGAAGGCGCTTCGTTGGAAGACTTTACGGAAGCGTTACGGATTGCTTTTAAAGAGGACACAGCGGTTTTAATTGAAGAGTTTTTACCTGGAACAGAATATCGGTTCTTTGTGTTAGATAATGATGTAAAAGCCATCATGTTGCGCGTGCCAGCCAATGTTACCGGAGATGGCAAACACACTGTAGAAGAATTGGTGGCCGCTAAAAATAGTGATCCATTGCGGGGGACCAATCACCGTGCACCACTAGAATTAATCCAGTTAAATGATTTAGAAAAACTAATGTTGAAAGAACAAGGTTTAACTATCTATTCTGTGCCAGAAAAAGAGCAAATCGTGTACTTGCGAGAAAATTCTAATGTTAGCACGGGCGGGGATTCGATTGATATGACCGATGTCATTGATGATAGTTATAAACAAATCGCCATTGAGGCCGTAGCTGCTTTAGGAGCCAAAATTTGTGGCATTGATTTAATCATTCCTGACAAAGACGTAAAAGGCACACGTGATAGCTTAACGTACGGGATTATCGAAGCAAACTTTAATCCAGCCATGCACATGCATGTGTATCCATACGCTGGACAGGGTAGACGCTTGACAATGGACGTTTTAAAACTTTTATACCCAGAAGTGGTTCAATAA |
| >gshAB_Efaeci | ATGATGAATTTTAAGCAATTATTATTGCATGACAATGCGCGTCCTTTTATCGACCAAGCTCGATTTGGTATAGAACGGGAAGGACAGCGGGTTGATCTTGCAGGAAATCTAGCAAAAACCGATCATCCAGCAATCTTTGGCGATCGATCCTATCATCCCTATATCCAAACAGATTTTAGCGAAACACAAACAGAGATGATCACCCCTGTTACCGATTCTATTCCCGAATTATTTCAGTATCTGGCTGCTGTTTATGATGTGACTGCTCGTTCTATACCGAAAGAAGAGATGATCTGGCCATTAAGTATGCCACCTGCCTTACCGGAAAAAGACGAAGAAATCATTATTGCAAAATTAAAAAATTTCGAAGATGTCTTGTATCGACGTTATTTAGCAAAAGAATATGGGAAGCGAAAACAGATGGTAAGCGGCATCCATTTCAATTTTGAATTTGGTGACGAATTATTAAGAACATTGTTCAGCCATCAGGAAGAATTTCAGGATTTTTCTGAATTCAAAACAGAACTTTATTTGAAAACAGCGAGAAATTTTTTACGTTATCGCTGGATGATCACTTATTTATTTGGTGCTTCACCGATGAGTGAGAAAAACTATTTTATAAATGAATCGCATCCGCAAGAACCTGTTCGCAGTATTCGAAACAGTGCGCTAGGCTACACGAATCATCCAAATGTGAAAGTTTCCTATGCTTCCATGAAACAGTATTTAGCAGATATTGAGCGAATGATCGAAGAAGGGAAACTTTCAGAGGAAAAAGAATTTTATACACCGCTTCGTTTCCGAGGCGGAAAGAAAGTCGCAGATTTAGCAACAACAGGCGTTCGTTATATTGAATTGCGGAATATCGATCTGAACCCTTACGCAAGGCTGGGAATCAATCCGGAACAAGTTCGTTTCTTACAATTATTCCTGATGTATATGCTATGGACAGAAGAAAAAGAAGACAGTGACCAATGGGTGGCAGAAGGTACAACTCGAAATAACAAAGTAGCACTAGAACAACCATCAGATCAAACAGAATTTCATCAAGAAGGTAGAGAAATCCTTGAAGGGATGAAGCAAATGTTGGTTGAATTGGATTGGTTGGATTCTCTTTATTTAGTGGAGGAAGCATTGATTCAAATGGATCATCCCGAACAAACATTAGCAGCGAAACTCTATCAAGAAGCACAGCTATCGAGTCAGCAAGAAGTTGCTGTGGCATTGGGGCATCAATATTATAAAGAAAGCCATGAACGTCCCTATCAGTTGGCTGGTTTTCGCGAAATGGAACTTTCTACTCAGATATTTATGTTCGATGCTATCCAAAAAGGTGTCCAAGTCAAAGTATTAGATGAGTCGGATCAATTTTTGCGTCTGCAATTCCAAGACCATGTAGAATATGTGAAAAATGCAAACATGACAAGTAAGGACAGCTATATCGTTCCACTTATCATGGAAAACAAAACAGTAACGAAAAAAGTTTTAAAAGAAGCTGGGTTCCGAGTGCCAGGTGGTGCAGAATTTTCATCCATGGAAGAAGCCGTAAAAGCTTATCCAAGATTTGCCGATCAGGCCTTTGTGATCAAACCTAAGTCCACAAATTACGGGTTGGGTATCACGATTTTCAAGGAAGGGGCTGATCTGGAGGATTATCAAGCAGGATTGGCGATTGCTTTTCGTGAAGACAGCTCGGTTTTAGTAGAAGAATTTATGCCGGGAACAGAATATCGTTTCTTTGTAATCGATGGAGAAGTCCAAGCCATCATGTTGCGAGTCCCTGCTAATGTTATTGGTGACGGTATACGAACAGTGAAAGAACTTGTTGAAGAAAAAAATAGTGACCCTTTGCGTGGAACGAATCATCGTGCACCGTTGGAATTGATCCAATTAGGCGAATTAGAACAATTGATGTTGAAAGAACAAGGGTTAACGATCGAATCAGTTCCCCAAGCCAATCAAATTGTTTATCTAAGGGAGAACTCAAATATCAGTACGGGCGGTGATTCGATCGATATGACTGATGAATTTTCTGAAACTTACAAAAAAATCGCCGTTTCTGCGGTAGAAGCACTAGGAGCTAAAATCAGTGGTATTGATTTGATCATACCAGATAAAGAAATCGATCCTACAACCGATAAAAAAGCATACGGGATCATCGAAGCAAATTTCAATCCAGCGATGCATATGCATGTTTATCCTTTTGCAGGAAAAGGCAGAAGATTGACGATGAATGTATTGAAATTGTTATATCCTGAAGTATTTTAA |
| >gshAB_Lc | ATGTTGAATCATTATTGGCAACTGATCCAGAAAAATAACTTGTTTGGCTTACGGGACGACACTCGGATTGGGGTGATTCGGACGGTGAAGCCGGTGTTGGCGGAAGGCGAGCAGCTGCCAGTGTGGGCGAATGCGAGTCGGCATGCGTTGGTGCAGCAGGTGTCGGCTACAATGTTACAGTTGACGACACCGCTTTTAGACGATCCCAGCGAAGTTGTGGCGTATTTGCGTGCGGGACTCAGCCGCGTCTGGGCAGCGCTGCCAGAAAACGGCAAGCTGCCGGTTTTCACCGCCGGATTGGAAAATAATCAGCGCATTCGCATTGTTTTGCCGGAAGTGTTGTTTGAGCGGCTTTATACGGCGGAAAATTTTCAAGGGAATGGCCCTGATTATGTGACGTACCGGAATCAAATTTATGATTTGGTCGCTGATGGGTTGATCACACAATTGCCGTTACTGACTTATCTCTTTGCCGCTTCACCGGCCAAAAACGGCCGGGCATTTTGGCCTGCGCCGGATCAAGAACGACAACTGGTTCAAAAGGTCAGTGACCCTGATCACTTAGCGCGAACATTGGCTTTGGATGTTGCAGTGGAACTTGATCCTTATGCGGCGAGCGGTGTTACGGAACAAATGTTGAACTTTTTAATCGACAGTTGCTGGTTTGCTTTGAGTCAACCAGCGATTCCATTGCCCGCGGCGGCTGAGGTGCGTCGGCATAGTTTGGCAGAAGCACAGAAGATTGCTGCTGGCGATCCCACGGCACCAGTCGCGAGTTTGACAGCACCGATTGATGCCATGGCGGTCTGGCTGAATCAGGTCGGCCTCACACCGCAACGCAAACAAGCTTTTGAGTTGATGCAAAGCCGAGTCCTGAAGCCAGAAACGACGATTGCGGGTCAGGTGGCGGCGGCTTATCATGATATTTCAGCAGCTCAAACACCGTTAGCAACGCAAGCCCACGCAGATTTGTCAATGGAAGAAGATTTACCGGGCTTCAGTAACTTGTCAGGCAACAGTCAAGCCTTGCTTCAGGCAGTGATTGATGGCGGGTATCAGTGGACCTTGCTGGATCGGGAACAGAACATTCTGCAGATTGCCAGTGATACTCAGCGCCATGTGTTGATTGACGGTGCGTTGACTAGCCGCACACCTGCTAGTGCCATGGTGGTAGCAGAACATCGCCATGCTGCCAAAAAGGTGTTAGCGGCGGCTGGGTTACCTGTTGCCAGAGGCGCCAAGTTCACACGTTGGCCTGAAGCTAAAGCTGCTTTTGAGCAAAGCTTTGCCCGTAAAAGTATCGTCGTGAAACCTGAACAGCGTAGTCATGGGCTTGCTGTTGAGCAATTTGCGGTGCCGCCAACTGCCAAACAATTCGCCCAAGCCTTTCACGCTGCTAACCAAGATCACGGCGTATTGGTCGAGATGATGGGGCGGGGAACAACGTATCATTTTACTGTTATAGGTCGCCGTGTGGTAAGTGTGTTGGAAAATGCCGCTGCCAATGTGGTTGGCGATGGTCGTAAATCAATCAAAGAATTGATTGCGTTGAAAAATGGTAAGCGGCCGAATGCACGGCAGCTCAAACTTGACGAAACAGCAAATCGGCAGTTGAAGTTACAATCGGTTACGATGAATACTGTCTTGCGGCGCGGTCAGCAGATTTTCCTTGCGTCTGCGGCGCATCCGCAAACAGGTGGCGATATTTATGATGTTACAACCGAAATCGATCCAAGCTACAACCAACTTGCGGTTGCCGCTGCTGATGCGCTTGAATTGCCGATTGCCGCCGTTGATATCGTGATTGATAATCTTTATGACGCCTATGCTGCTGAGCATGAAGGCCAAGCTATCATCATCAGTGTCGATCCGATTCCCGACTTAATGTTGCCACAACAACCAGATATGGGCGCGGCGCATTCAATTGCACCTGCTTTACTCACGTATCTGTTTAGCGAAAAATAA |
| >gshAB_Lpara | ATGTTGAATCATTATTGGCAACTGATCCAGAAAAATAACTTGTTTGGCTTACGGGACGACACTCGGATTGGGGTGATTCGGACGGTGAAGCCGGTGTTGGCGGAAGGCGAGCAGCTGCCAGTGTGGGCGAATGCGAGTCGGCATGCGTTGGTGCAGCAGGTGTCGGCTACAATGTTACAGTTGACGACACCGCTTTTAGACGATCCCAGCGAAGTTGTGGCGTATTTGCGTGCGGGACTCAGCCGCGTCTGGGCAGCGCTGCCAGAAAACGGCAAGCTGCCGGTTTTCACCGCTGGATTGGAAAATAATCAGCGCATTCGCATTGTTTTGCCGGAAGTGTTGTTTGAGCGGCTTTATACGGCGGAAAATTTTCAAGGGAATGGCCCTGATTATGTGACGTACCGGAATCAAATTTATGATTTGGTCGCTGATGGGTTGATCACACAATTGCCGTTACTGACTTATCTCTTTGCCGCTTCACCGGCCAAAAACGGCCGGGCATTTTGGCCTGCGCCGGATCAGGAACGGCAACTGGTTCAAAAGGTCAGTGACCCTGATCACTTAGCGCGAACATTGGCTTTGGATGTTGCAGTGGAACTTGATCCTTATGCGGCGAGCGGTGTTACGGAACAAATGTTGAACTTTTTAATCGACAGTTGCTGGTTTGCTTTGAGTCAACCAGCGATTCCATTGCCCGCGGCGGCTGAGGTGCGTCGGCATAGTTTGGCAGAAGCACAGAAGATTGCTGCTGGCGATCCCACGGCACCGGTCGCGAGTTTGACAGCACCGATTGATGCCATGGCGGTCTGGCTGAATCAGGTCGGCCTCACACCGCAACGCAAACAAGCTTTTGAGTTGATGCAAAGCCGAGTCCTGAAGCCAGAAACGACGATTGCGGGTCAGGTGGCGGCGGCTTATCATGATATTTCAGCAGCTCAAACACCGTTAGCAACGCAAGCCCACGCAGATTTGTCAATGGAAGAAGATTTACCGGGCTTCAGTAACTTGTCAGGCAACAGTCAAGCCTTGCTTCAGGCAGTGATTGATGGCGGGTATCAGTGGACCTTGCTGGATCGGGAACAGAACATTCTGCAGATTGCCAGTGATACTCAGCGCCATGTGTTGATTGACGGTGCGTTGACTAGTCGCACACCTGCTAGTGCCATGGTGGTAGCAGAACATCGCCATGCCGCCAAAAAGGTGTTAGCGGCGGCTGGGTTACCTGTTGCCAGAGGCGCCAAGTTCACACGTTGGCCTGAAGCTAAAGCTGCTTTTGAGCAAAGCTTTGCCCGTAAAAGTATCGTCGTGAAACCTGAACAGCGTAGTCATGGGCTTGCTGTTGAGCAATTTGCGGTGCCGCCAACTGCCAAACAATTCGCCCAAGCCTTTCACGCTGCTAACCAAGATCACGGCGTATTGGTCGAGATGATGGGGCGGGGAACAACGTATCATTTTACTGTTATAGGTCGCCGTGTGGTAAGTGTGTTGGAAAATGCCGCTGCCAATGTGGTTGGCGATGGTCGTAAATCAATCAAAGAATTGATTGCGTTGAAAAATGGTAAGCGGTCGAATGCACGGCAGCTCAAACTTGACGAAACAGCAAATCGGCAGTTGAAGTTACAATCGGTTACGATGAATACTGTCTTGCGGCGCGGTCAGCAGATTTTCCTTGCTTCTGCGGCGCATCCGCAAACAGGTGGCGATATTTATGATGTTACAACCGAAATCGATCCAAGCTACAACCAACTTGCGGTTGCCGGTGCTGATGCGCTTGAATTGCCGATTGCCGCCGTTGATATCGTGATTGATAATCTTTATGACGCTTACGCTGCTGAGCATGAAGGCCAAGCTATCATCATCAGTGTCGATCCGATTCCCGACTTAACGTTGCCACAACAACCAGATATGGGCGCGGCGCATTCAATTGCACCTGCTTTACTCACGTATCTGTTTAGCGAAAAATAA |
| >gshAB_Lplant | ATGGAATTAGATGCCGTTGGTAAGGCAATTGTACAGTATCACTTAGTCCCACTCGTTCATCAGGCTAATTTAGGACTAGAGGTCACCATGCACCGGGTGGACGACCATGGTCACTTAGCGACGACAGCACACCCGCAAGCGTTTGGATCAGCGCAACAAAATCATCAGTTACGTCCGGGCTTTTCCGCAAGTGCTTTAAAGTTTACTACGCCGGTGCGTCGTGACATTCCTGCATTGATGGCGTATCTGAAGGGCTTGAATACCGCAGCACGGCGGTCACTCGATGCGGACGAACGACTTTGGCCACTGTCGAGTACGCCTGTGTTGCCGGATGATCTAACGAACGTGCCACTGGCTGATGTTGATCAAGTCAGCTATCAGCGTCGTCGCGACTTAGCTCGTAAGTATGAGTTACAGCGATTAATGACGACTGGTAGTCACGTGAATATGAGCTTGAATGAAGCTTTATTCACCCGTTTATATACTGAGACTTTCCATCAGCAGTATCACAGTTATGTTGACTTTCGCAATGCAATTTATCTGAAAGTCGCTCAGGGATTGGTGCGCATGAACTGGCTGATTCAGTATTTATTTGGCGCTTCACCACGCCTAGCCGTTACGGATACTACGAGTCGTCCACAGCGCAGTAGTGTTCAACATGGTCGCTACAGTCAAGTGACGGGAGACTATACGTCAATTGATCGCTACGTGGCCAAGTTGACGGCGGCTGTTCGTCAACAGCAGTTGTTGTCGGTCAATGATTTTGACGGGCCAGTTCGGCTTCGGAGTAATGGGCAGCTAGCTATGATGGCCCGGCAGGGGGTCTATTATCTTGAATACCGGGGCTTGGATCTCGATCCAACTAGTCCAGTCGGGGTGGACGCGAACGCGGTGGCATTTGTTCGTTTGTTGGCGAGTTATTTCGTAATGATGCCGGCACTTCCAGCTAAGATGGTATCCCAAGTCAACGCTCAAGCTGACCAATTGACCCGTCAAGTTTTGGGTGAAAATCCAACGACGGCTAGTGCTCAGGCCGTGCCGGCTGTTCAAGTTTTAGATGCACTTGCTGATTTTGTTAAAACCTATGGCCTACCAAATGAAGATGCCGTGTTACTCAAACAGTTGAAGTCGCGGGTCACTGATCCAAAGAAGACGCTGAGTGCGCAGATTGCCATGCAAGCCGATCCGTTAGCATGGGCACTCGAACGGGCTGCACGCTATCAGGAATCGAGCAATGAACGTCCGTTTGAACTTGCGGGCTTTACCGCGCTAGATCTATCGAGCCAGCAACTAGCCCAGCAGGCCTTGACGCGGGGAGTGCAGGTGGACGTTGTTGACCCACACGCTAACATTTTACGATTGACTAAGTTAGGACGGTCGCAATTAGTTGTGAATGGGAGCGGAACGGATTTAAATCCACAGGCGCTAACGACCGTACTGACACATAAAGCAGCGGCCAAACAAATTCTGGCTGAGCACGGGGTTCCGGTGCCGGCTTCACAGACATATCATACAGCTAATCAGTTGATTGCTGATTATGATCGATACGTTCAAGCTGGTGGGATCGTATTAAAAGCGGCGGATGAGTCGCACAAAGTAATTGTCTTTCGGATTATGCCCGAACGCGGACTGTTTGAACAAGTCGTCCGGCAACTATTCGAGCAAACGTCCGCGGTAATGGCCGAGGAAGTGGTAGTCGCATCAAGTTATCGCTTTTTGGTTATCGATGGTCGTGTGCAAGCAATCGTCGAACGAATTCCAGCCAATATTGTTGGTGATGGTCGCTCAACGGTCAAGACGTTACTTGATCGCAAAAATGGTCGAGCGTTGCGCGGGACCGCTTTTAAGTGGCCTCAATCAGCGCTACAGTTAGGAACGATCGAACGGTATCGCCTGGACTCATATCACTTGACCTTAGATTCTGTGGTCAGCCGGGGAACTCAGATCTTATTACGAGAGGATGCGACTTTTGGTAACGGGGCGGACGTGCTAGACGCGACGGCTGATATGCATCAATCCTATGTGCAGGCGGTGGAAAAGTTGGTAGCAGACTTACACTTAGCGGTCGCTGGGGTCGACGTGATGATTCCCAATCTCTATGCCGAATTAGTGCCAGAGCATCCTGAAATGGCGGTATACTTGGGTATTCATGCGGCGCCGTACTTGTATCCGCACTTGTTCCCAATGTTTGGTACTGCCCAACCAGTGGCGGGGCAGTTGTTGGATGCATTGTTTAAAAATGAAGATTAA |
| >gshAB_Lr | ATGAATCATTATTGGCAACTGATTCGAAAAGAACATTTGTTTGGCTTGGCCGCTCAAACGCGAATGGGTGTGATTCGAACTGTCAAACCCATTCTGCCGGCAGAAACCAACTTACCGCAGTGGCCGCAACGTAGCCGCCATGCTTTGGTTCAGCCACTTTCTGCTTCAGTGTTGCAAATCGTAACCCCATTACTAGACGATTTTCCTGCCGTTATTGACCAGTTACGGGCTGGGCTAAGTCGAATTTGGACGGCATTACCGGAAGCAGGGCACTTATCGGCTTTTACCGCGGAAAAAGTGAACCAGCAAACGGTCCGGATAGTTTTACCGGAAACACTATTTGAACGGTTGTACACAGCAGAGAATTTTCAGGGCCATGGTCCGGAATATGTTGCCTATCGTAATGAAGTTTATCAGTTGGTAGCAGACGGGTTAACGCGGCAGTTGCCATTGTTGACTTACTTGTTTTCTGCTTCTCCAGCTGACGGTCACCGCGCATTTTGGCCGTCACCGGATCAGGAACGGCAACTGGTTCAAAAAGTGACGGATCCAGACCACTTGGATCGAATTCTTGCGCTGGACGTTGTGGTGGAGCTTGATCCGTACGCTGCTAGCGGGATCACGACCCAAATGCTGACATTCTTGCGTGATAGCTGCTGGTTTGCGCTAAGTCAACCGGCCATTCCGGTTGCCGCGGCAAGTGAAGTACGCCGGCATGCTGTCATGCAGGCGCGGACAATTGCCCAGATGGCACCTGATGCCAAAGTCGCCGAGCTGCCGGCAATGCTGGATGCGATGGCGACATGGTTAAATCATGTTGGCATTGATGCGCAACAAAAACAAGCTTTTGAATTGATGCAGACGCGATTAGTGAAGCCGGAAACGAGCCTTGCCAGTCAAGTTCTTGCAACGTTTCATGACCAAACAGCTGCGGATGAGTCATTGAGTCGGCAAGCACAGGCGGACTTAACCAGTACCAATGAGTTGCCGGGGATGACAGATTTATCGGAAAACACCCAGACATTGTTACAAGCTGCTATGGATGGGGGGTATCAGTTCACGATACTGGATCGTCAACAGAATCTGGTGCAAATTGCCACGGATACCCAGACACAAGTCATTGCAGACGGCGTTGTTACCAACTATACGCCAGCCAATGCCATGATCGTTGCCACCCATCGGCACACAGCCAAACAGTTGCTGGCCGCAGCAGGAATACCAGTTGCACGTGGGGCTAAGTTTACTAAATGGCCGGATGCCAAAGCAGCTTTTGAGCACAGCTTTGCGCATAAAAGTATTGTGGTGAAACCCGAGGCACGCAGCCAAGGCGAAGCGGTTGAGCAGTTTTCGATACCACCGACTGAAAAGCAGTTTGACCGAGCCTTTCATGAAGCCAATCGCCATCATGGGGTGCTCATTGAAATGATGGCACGCGGCACGACCTACCATTTTACCATCATCGGGCAACAAGTGCTCAGCGTTTTGGAAACAGCAGCAGCTAATGTTGTAGGCGATGGGCGCAAAGCCATTAAGGAATTGATCGCCTTGAAAAATGGTCACCGCGCGACTTCCCGGCAATTGCAGCTTGATGCTAGCGCGCGCCGTCAGTTAAAGGCACAAGCGTTGACACCTGAGACTGTGCTTCAACGCGGGCAGCAGGTTTTCTTAACCACTGCCGCGCATCCGCAAACCGGTGGCGATTTGTATGACGTGACGGACGAGATTGATGACAGTTACAAGCAACTGGCGCTAAAAGCTGCCGCCACACTTGATTTGCCGGTAGCAGCTGTCGACATTGTGATTGATAATCTGTATGCACCATATGATCCGGAGGCAGATGGGCAGGCGAACGTAATTAGTGTCAATCCGGTACCGGATCTCGCTGCGCCTTTGCACCTGGACATGGGCGAATCACGCGCACTTGCCCCGGCATTGCTAAACTGGCTGTTTGCTGTGAGATAA |
| >gshAB_St | ATGACATTAAACCAACTTCTTCAAAAACTGGAAGCTACCAGCCCTATTCTCCAAGCTAATTTTGGAATCGAGCGCGAGAGTCTACGTGTCGATAGGCAAGGACAACTGGTGCATACACCTCACCCATCCTGTCTAGGAGCTCGTAGTTTCCACCCCTATATTCAGACTGATTTTTGCGAGTTTCAGATGGAACTCATCACACCAGTTGCCAAATCTACTACTGAGGCTCGCCGATTTCTGGGAGCTATTACTGATGTAGCAGGCCGCTCTATTGCTACAGACGAGGTTCTCTGGCCTTTGTCCATGCCACCTCGTCTAAAGGCAGAGGAGATTCAAGTTGCTCAACTGGAAAATGACTTCGAACGCCATTATCGTAACTATTTGGCTGAAAAATACGGAACTAAACTACAAGCTATCTCAGGTATCCACTATAATATGGAACTGGGTAAAGATTTAGTTGAGGCCTTGTTTCAAGAAAGTGATCAGACCGATATGATTGCCTTCAAAAACGCCCTCTATCTTAAGCTGGCTCAGAACTACTTGCGCTACCGTTGGGTGATTACCTATCTCTTTGGGGCCTCACCCATCGCCGAACAAGGTTTCTTTGACCAGGAAGTTCCAGAACCTGTGCGTTCCTTCCGTAACAGTGACCACGGCTATGTCAATAAGGAAGAGATTCAAGTATCCTTTGTAAGTCTAGAAGATTATGTCTCAGCCATTGAAACCTATATCGAACAAGGAGATTTGATTGCAGAGAAAGAATTTTACTCAGCTGTTCGTTTCCGTGGACAAAAGGTTAATCGTTCCTTCCTTGACAAGGGAATCACCTACCTAGAGTTCCGTAATTTCGACCTTAATCCTTTTGAGCGTATCGGTATTAGTCAGACTACTATGGACACTGTGCACTTACTCATTTTAGCCTTCCTTTGGCTTGATAGCCCTGAAAATGTCGACCAAGCTCTTGCACAAGGCCACGCGCTAAATGAGAAAATTGCCCTCTCTCATCCTCTAAAACCTCTACCTTCGGAGGCTAAAACTCAGGACATTGTAACTGCCCTAGACCAACTGGTGCAACACTTTGGACTTGGTGACTATCATCAAGATCTGGTTAAACAAGTTAAGGCAGCCTTTGCGGATCCAAATCAAACGCTCTCTGCCCAGCTCTTACCCTATATCAAAGACAAATCTCTAGCCGAATTTGCTTTAAACAAGGCTCTTGCCTATCATGATTACGACTGGACTGCCCACTATGCTCTCAAGGGCTATGAAGAGATGGAACTCTCCACCCAGATGTTGCTCTTTGATGCCATCCAAAAGGGGATTCACTTTGAAATATTGGATGAGCAAGATCAATTCCTAAAACTTTGGCACCAAGACCATGTTGAATACGTCAAAAACGGTAACATGACCTCAAAAGACAACTACGTGGTTCCCCTTGCTATGGCTAATAAGACCGTAACCAAGAAGATTCTAGCAGATGCTGGCTTTCCAGTTCCTTCAGGAGACGAATTTACCAGTCTTGAGGAAGGACTTGCCTACTACCCTCTTATCAAGGATAAGCAAATTGTTGTCAAACCCAAGTCAACTAACTTTGGTCTGGGAATTTCCATTTTCCAAGAACCTGCCAGTCTTGACAACTATCAAAAAGCCCTTGAAATTGCTTTCGCAGAAGATACCTCTGTCCTTGTTGAAGAATTTATTTCAGGAACCGAATACCGTTTCTTCATCTTGGATGGGCGTTGTGAGGCTGTGCTTCTGCGTGTCGCTGCCAATGTTATTGGTGATGGCAAACACACCATTCGTGAACTAGTCGCTCAGAAAAATGCTAATCCATTGCGTGGCCGTGATCACCGGTCACCTCTGGAAATCATTGAGCTAGGAGACATCGAACAACTAATGTTAGCTCAACAGGGTTATACACCTGATGATATTCTCCCAGAAGGAAAAAAGGTCAATCTGCGTCGTAATTCCAACATCTCTACAGGTGGTGACTCTATTGATGTCACTGAGACCATGGATTCCTCTTACCAAGAATTAGCCGCAGCCATGGCAACTAGCATGGGCGCCTGGGCTTGCGGGGTTGATCTGATAATTCCAGATGAAACTCAAATTGCCACCAAGGAAAATCCTCATTGCACCTGCATTGAGCTCAACTTTAACCCTTCGATGTATATGCACACCTACTGTGCTGAGGGTCCTGGCCAAGCTATCACTACTAAAATCCTAGATAAACTTTTTCCAGAAATAGTGGCTGGTCAAACTTAA |
| >gshAB_St(Codon-op) | ATGACTTTGAACCAATTGTTGCAAAAGTTGGAAGCTACCTCTCCAATTTTGCAAGCTAACTTCGGTATCGAACGTGAATCCTTGAGAGTTGATAGACAAGGTCAATTAGTTCACACCCCACATCCATCATGTTTGGGTGCTAGATCTTTCCATCCATACATTCAAACTGACTTCTGTGAATTCCAAATGGAATTAATCACCCCAGTTGCTAAATCTACTACCGAAGCTAGAAGATTTTTAGGTGCTATTACTGATGTTGCAGGTCGTTCTATTGCTACTGATGAAGTTTTGTGGCCATTGTCAATGCCACCTAGATTGAAGGCTGAAGAAATTCAGGTTGCTCAATTAGAAAACGATTTTGAAAGGCATTATCGTAACTATTTGGCTGAAAAGTACGGTACCAAGTTACAAGCTATTTCCGGTATTCACTATAATATGGAATTAGGCAAGGATTTGGTTGAAGCATTGTTCCAAGAATCTGACCAAACTGACATGATCGCTTTCAAGAATGCTTTGTACTTGAAATTAGCTCAAAATTACTTAAGATACAGATGGGTTATTACCTACCTATTCGGTGCTTCTCCAATCGCTGAACAAGGTTTCTTCGATCAAGAAGTCCCAGAACCAGTTAGATCTTTCAGAAACTCTGACCATGGTTACGTCAACAAAGAAGAAATTCAAGTTTCTTTCGTTTCCTTGGAAGATTACGTGTCAGCTATTGAAACATACATTGAACAAGGCGATTTGATTGCTGAAAAGGAATTCTATTCTGCTGTTCGTTTCAGAGGTCAAAAAGTTAACAGGTCTTTCCTAGATAAAGGTATTACTTACCTGGAATTCAGAAACTTTGATTTGAACCCTTTTGAAAGAATTGGCATTTCTCAAACTACTATGGACACAGTTCACTTGTTGATTTTGGCTTTTCTTTGGTTGGATTCTCCTGAAAACGTTGATCAAGCTTTAGCTCAAGGTCATGCTTTGAATGAAAAGATTGCTTTGTCTCATCCTTTGAAGCCATTACCATCTGAAGCTAAGACTCAAGATATTGTCACCGCACTAGACCAATTGGTCCAACACTTCGGTTTGGGTGACTATCATCAAGATTTGGTTAAACAAGTTAAAGCTGCCTTTGCTGATCCTAACCAAACCTTGAGTGCTCAACTTTTGCCATATATCAAGGATAAATCTTTAGCTGAATTCGCCTTGAATAAGGCCCTTGCATACCATGATTACGACTGGACAGCACATTATGCTTTGAAAGGTTACGAAGAGATGGAATTGTCTACTCAAATGTTACTATTTGATGCTATTCAAAAGGGAATTCATTTCGAAATTCTAGATGAACAAGATCAATTCCTGAAGTTATGGCACCAAGACCATGTTGAATACGTTAAGAACGGTAACATGACTTCCAAGGATAATTACGTTGTTCCATTAGCTATGGCTAATAAGACCGTTACCAAGAAAATTTTAGCTGATGCTGGTTTTCCAGTTCCATCAGGCGATGAATTCACATCCTTAGAAGAAGGTTTAGCCTACTATCCATTGATCAAGGACAAACAAATTGTCGTTAAACCAAAGTCTACAAATTTCGGTTTAGGTATTTCCATTTTTCAAGAACCAGCTTCTCTAGATAACTATCAAAAGGCCTTGGAAATCGCTTTCGCCGAAGATACCTCAGTTTTAGTCGAAGAGTTCATTTCTGGTACTGAATACAGATTCTTCATCTTGGATGGTAGATGTGAGGCTGTCTTATTGAGAGTTGCTGCCAATGTTATTGGAGATGGTAAACATACTATTAGAGAATTGGTCGCCCAAAAGAACGCTAACCCATTAAGAGGTCGTGACCATAGGAGTCCATTGGAGATCATTGAATTGGGTGACATTGAACAATTGATGTTGGCTCAACAAGGTTACACTCCTGACGATATTTTGCCTGAAGGTAAAAAGGTTAATCTAAGAAGAAATTCTAACATTTCCACCGGTGGCGATTCTATTGATGTAACTGAAACCATGGATTCCTCTTACCAAGAATTAGCTGCTGCTATGGCTACTAGCATGGGTGCTTGGGCTTGTGGTGTTGACTTGATTATCCCAGATGAAACTCAAATTGCTACTAAGGAAAATCCACATTGTACTTGTATTGAATTGAACTTTAATCCATCTATGTACATGCACACTTATTGTGCTGAAGGTCCAGGTCAAGCTATTACTACTAAAATTTTAGATAAATTGTTCCCAGAAATTGTCGCTGGTCAAACTTAA |
